# Supplementary material for: Taenia solium excretory secretory proteins (ESPs) suppresses TLR4/AKT mediated ROS formation in human macrophages via hsa-miR-125
Source: PLoS Negl Trop Dis. 2023 Dec 29;17(12):e0011858. doi: 10.1371/journal.pntd.0011858 (PMC10783723; doi:10.1371/journal.pntd.0011858)
Supplement: S2 File — Fig A. Flow chart outlining the method. Fig B. Cysts infested pork from naturally infected swine (a) and isolated cyst (b). Fig C. Species identification of Taenia by PCR, all our samples were positive only for T. solium and gave 984bp product specific for this species. Fig D. SDS-PAGE and silver staining of T. solium ESPs. Fig E. Quantification of progression of secreted cytokines by ELISA. Fig F. Apoptosis assay. The ESP treated cells showed no sign of apoptosis as compared to positive control. Fig G. Caspase activity. The ESP treated cells showed no sign of caspase 3 as compared to positive control. Fig H. Titration of ESP to establish diminished TLR4 expression and simultaneously decreased phosphorylation of Akt. (DOCX) [file pntd.0011858.s002.docx]

**Title: *Taenia solium* excretory secretory proteins (ESPs) suppresses TLR4/AKT mediated ROS formation in human macrophages via hsa-miR-125**

Naina Arora^1^, Anand Kumar Keshri^1^, Rimanpreet Kaur^1^, Suraj Singh Rawat^1^, Rajeev Kumar^2^, Amit Mishra^3^, Amit Prasad^1*^

^1^School of Biosciences and Bioengineering, Indian Institute of Technology Mandi, Mandi, Himachal Pradesh, India

^2^CSIR-Institute for Himalayan Bioresource Technology, Palampur, Himachal Pradesh, India

^3^Cellular and Molecular Neurobiology Unit, Indian Institute of Technology Jodhpur, Rajasthan, India

* [amitprasad@iitmandi.ac.in](mailto:amitprasad@iitmandi.ac.in)

**Supplementary Information**


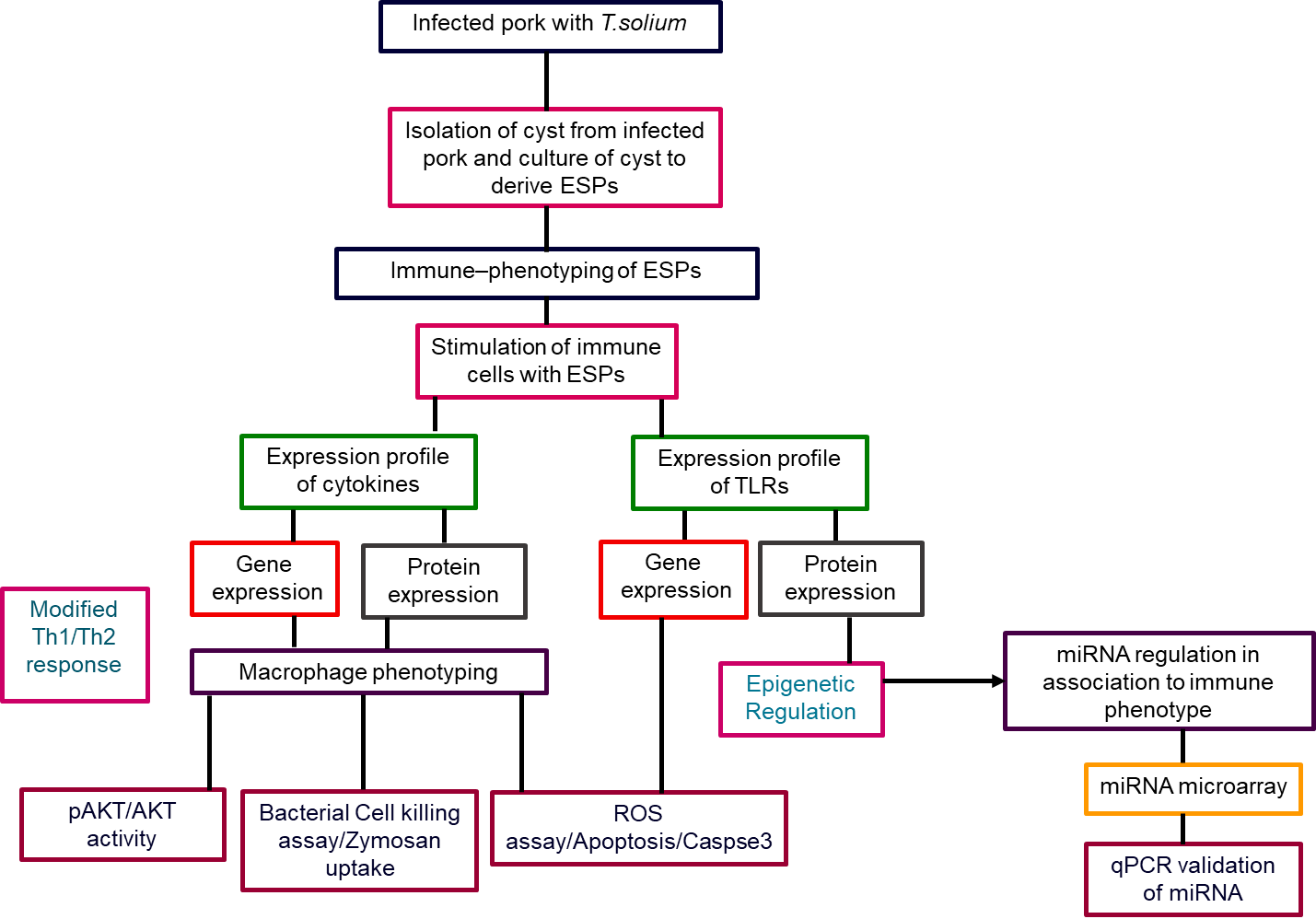


**Fig A.** Flow chart outlining the methodology.

- 1. **Isolation and culture of *T. solium* cyst:**

**
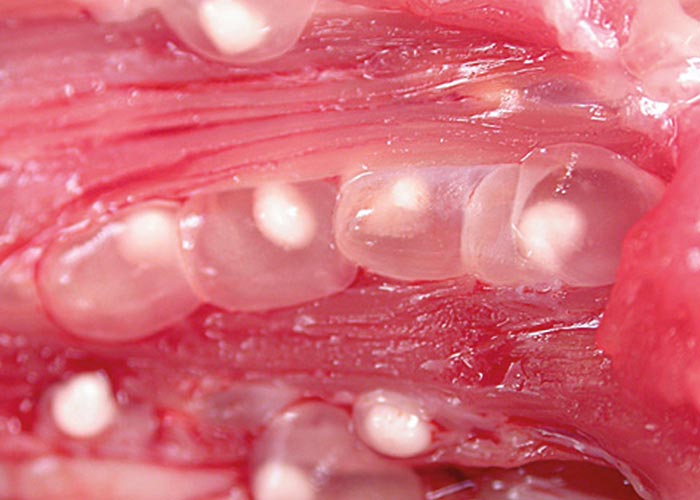

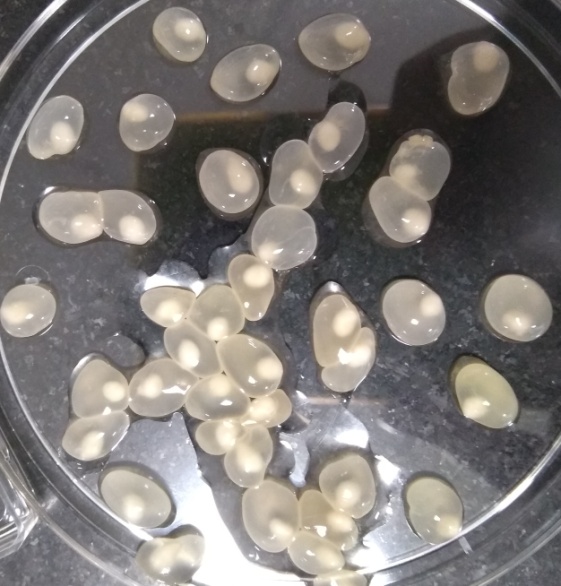
**

(a) (b)

**Fig B.** Cysts infested pork from naturally infected swine (a) and isolated cyst (b).

**1.2. Multiplex PCR for Taenia species identification:**

We first confirmed the Taenia speciation by using previously described method. Species identification was done by multiplex PCR for cytochrome c oxidase subunit 1 gene (cox1) on DNA isolated from the segments as described earlier (Yamasaki et al., 2004; Prasad et al., 2007). The PCR confirmed that this cyst isolated were of *T. solium* species only.


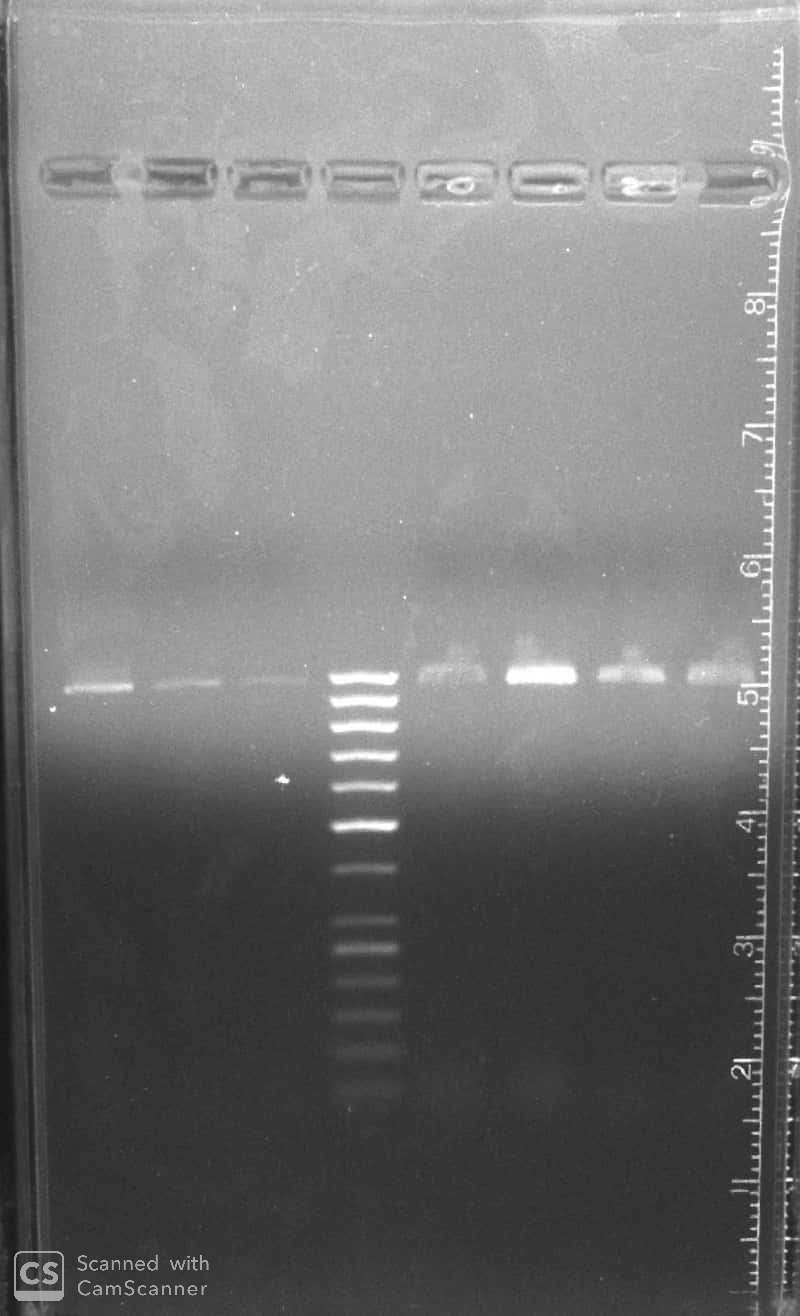


1

2

3

MW

**Fig C.** Species identification of Taenia by PCR, all our samples were positive only for *T. solium* and gave 984bp product specific for this species.

**1.3. SDS PAGE- Silver staining of ESPs**:

ESPs isolated in the previous step along with only RPMI and RPMI with 10% FBS were subjected to SDS-PAGE followed with silver staining for proteins following standard protocol. Silver staining identified a number of extra bands in ESPs present other than control (RPMI and RPMI with 10% FBS). Thus, confirming that *T. solium* cysts secrete large amount of antigens in its culture medium. (**Fig D**)


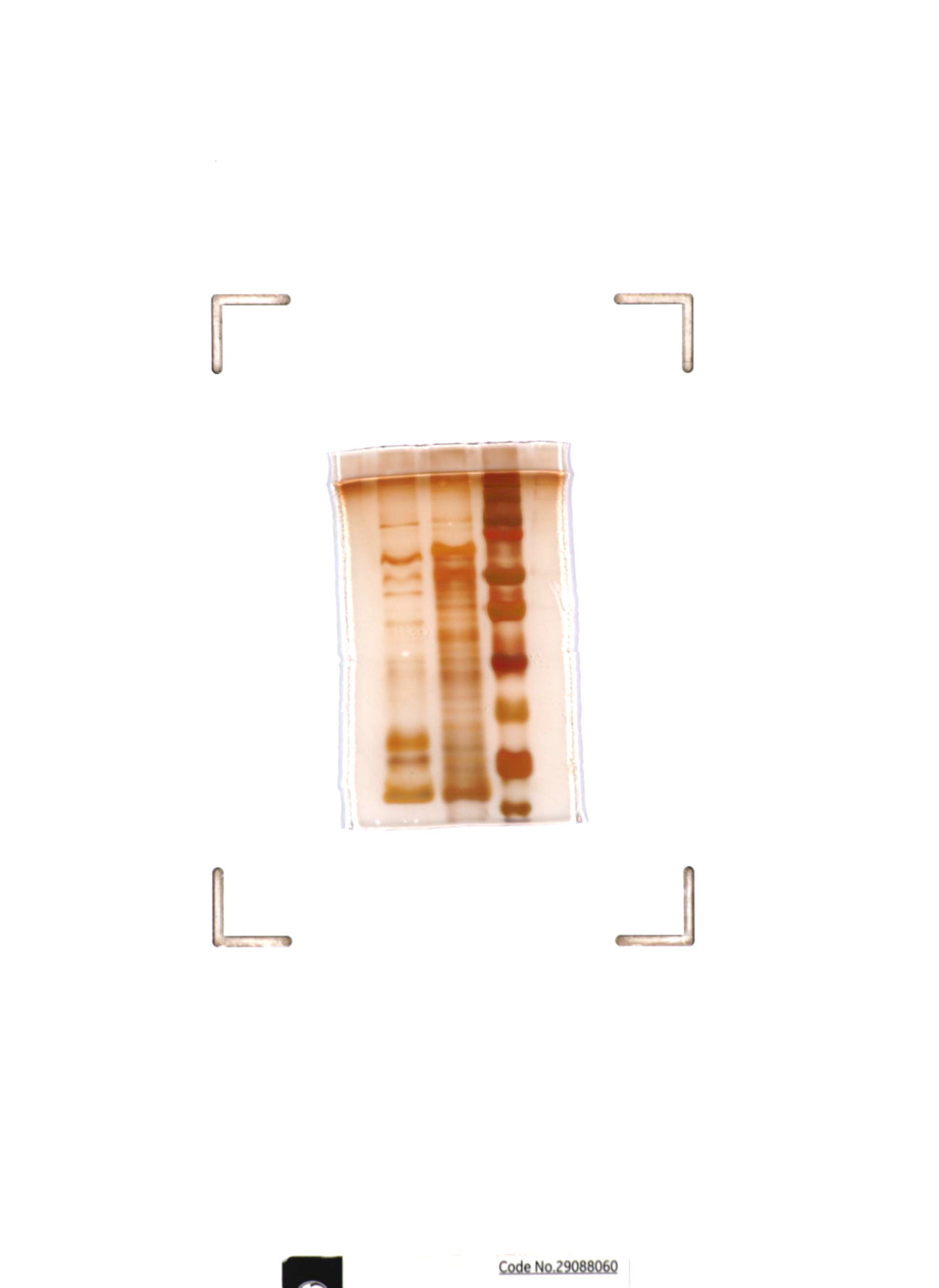


ESP M RPMI

**Fig D.** SDS-PAGE and silver staining of T. solium ESPs.


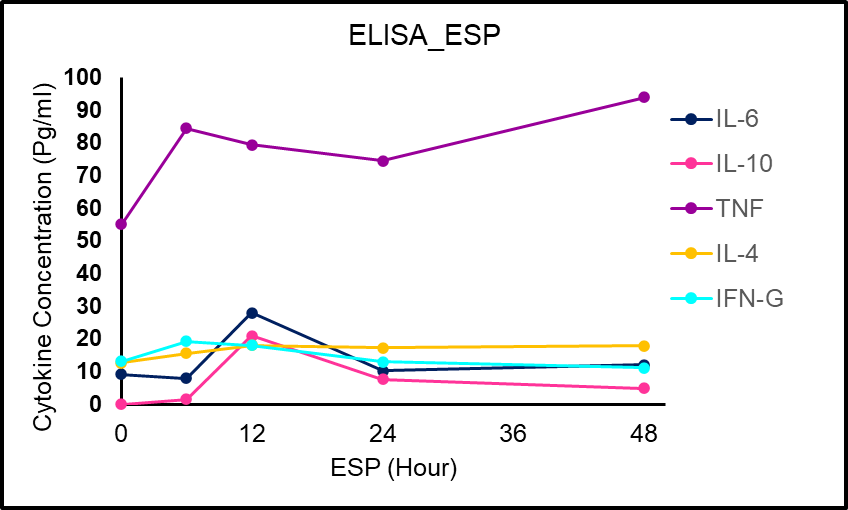


**0 6 12 24 36 48**

**Fig E.** Quantification of progression of secreted cytokines by ELISA.

**1.4. FITC Annexin V Apoptosis Assay**


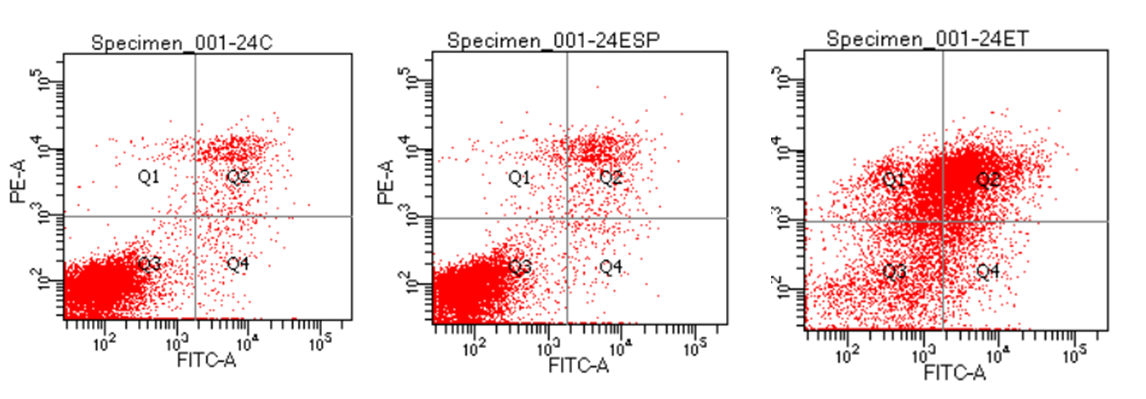
The apoptosis assay was carried using FITC Annexin V Apoptosis Detection Kit (#556547) as described by manufacturer. In brief, 1*10^6 cells/ml per treatment group, Etoposide 5uM was taken as positive control (Sigma) were taken, washed with PBS twice and re-suspended in 1ml of binding buffer. For staining the cells, 100ul of resuspension was taken in 5ml tube and 5ul of FITC and PI each were added to the tube, slightly vortexed and incubated for 15min at room temperature in dark. Post incubation 400ul of binding buffer was added to each tube and samples were acquired on BD LSR Fortessa Flow cytometer.

| Population | Control (%) | ESP(%) | Etoposide(%) |
| --- | --- | --- | --- |
| Q1 (Dead cell) | 1.1 | 2.2 | 21.8 |
| Q2 (Late apoptotic) | 7.2 | 8.0 | 42.5 |
| Q3 (Live Cell) | 89.1 | 87.8 | 26.1 |
| Q4 (Early apoptotic) | 2.6 | 2.0 | 9.6 |

**Fig F.** Apoptosis assay. The ESP treated cells showed no sign of apoptosis as compared to positive control.

**1.5. Human Caspase 3 activity assay:**

Caspase 3 activity was assayed using caspase ELISA kit (# KHO1091) as per the manufacturer’s instruction. In brief, 1*10^7 cells/ ml was used to prepare the cell lysate. 10ul of clear lysate post processing was diluted (1:10) and incubated in the pre coated well at RT for 2 hours, after incubation wells were washed and incubated with detection antibody for 1 hr at RT. Wells were washed post incubation and incubated with secondary antibody for 30 min at RT. After washing the wells, stabilized chromogen was added for 30 min at RT and reaction was terminated using stop solution and read at 450nm. The concentration was quantified against standard given with the kit.

**Fig G.** Caspase activity. The ESP treated cells showed no sign of caspase 3 as compared to positive control.

**
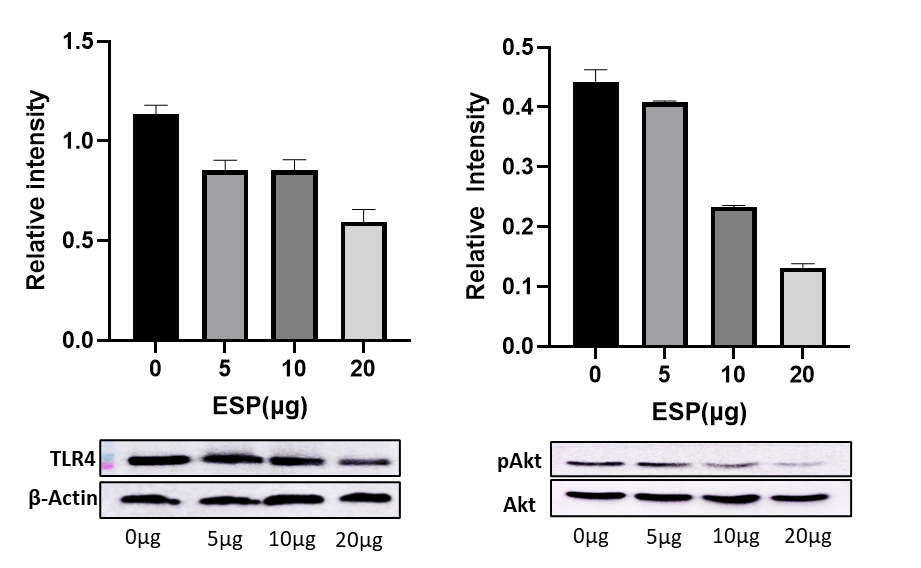
**

**Fig H.** Titration of ESP to establish diminished TLR4 expression and simultaneously decreased phosphorylation of Akt.
