# Supplementary material for: Taenia solium excretory secretory proteins (ESPs) suppresses TLR4/AKT mediated ROS formation in human macrophages via hsa-miR-125
Source: PLoS Negl Trop Dis. 2023 Dec 29;17(12):e0011858. doi: 10.1371/journal.pntd.0011858 (PMC10783723; doi:10.1371/journal.pntd.0011858)
Supplement: S1 Table — (DOCX) [file pntd.0011858.s003.docx]

**S1 Table: List of primers used in the study**

| **S.No** | **Target Gene** | **Forward Primer 5’-3’** | **Reverse Primer5’-3’** |
| --- | --- | --- | --- |
| 1 | IFN ϒ | TCGGTAACTGACTTGAATGTCCA | TCGCTTCCCTGTTTTAGCTGC |
| 2 | TNFα | GGAGAAGGGTGACCGACTCA | CTGCCCAGACTCGGCAA |
| 3 | IL1beta | ATGATGGCTTATTACAGTGGCAA | GTCGGAGATTCGTAGCTGGA |
| 4 | IL-2 | GTCACAAACAGTGCACCTAC | CCCTGGGTCTTAAGTGAAAG |
| 5 | IL-4 | CGAAGAACACCACAGAGACTGAGCT | GACTCATTCATGGTGCAGCTTATCG |
| 6 | IL-6 | GTAGCCGCCCCACACAGA | CATGTCTCCTTTCTCAGGGCTG |
| 7 | IL-10 | CATCGATTTCTTCCCTGTGAA | TCTTGGAGCTTATTAAAGGCATTC |
| 8 | TLR1 | TTCAAACGTGAAGCTACAGGG | CCGAACACATCGCTGACAACT |
| 9 | TLR2 | ATCCTCCAATCAGGCTTCTCT | GGACAGGTCAAGGCTTTTTACA |
| 10 | TLR3 | CAAACACAAGCATTCGGAATCTG | AAGGAATCGTTACCAACCACATT |
| 11 | TLR4 | AGTTGATCTACCAAGCCTTGAGT | GCTGGTTGTCCCAAAATCACTTT |
| 12 | TLR5 | CCGGGTTTGGCTTCCATAACA | TGTGAAAGATCCAGGTGTCTCA |
| 13 | TLR6 | TTCTCCGACGGAAATGAATTTGC | CAGCGGTAGGTCTTTTGGAAC |
| 14 | TLR7 | TCCTTGGGGCTAGATGGTTTC | GGTATGTGGTTAATGGTGAGGGT |
| 15 | TLR8 | GACTACAGGAAGTTCCCCAAAC | ATACCGGGATTTCCGTTCTGG |
| 16 | TLR9 | AATCCCTCATATCCCTGTCCC | GTTGCCGTCCATGAATAGGAAG |
| 17 | TLR10 | GATTTACTCTGGGACGACCTTTT | GTCAAGATAAGCCTTACCACCAA |
| 18 | β-Actin | TCACCCACACTGTGCCCATCTACG | CAGCGGAACCGCTCATTGCCAATG |
| 19 | GAPDH | ATCACCATCTTCCAGGAGCGA | CCTTCTCCATGGTGGTGAAGAC |
