# Supplementary material for: Taenia solium excretory secretory proteins (ESPs) suppresses TLR4/AKT mediated ROS formation in human macrophages via hsa-miR-125
Source: PLoS Negl Trop Dis. 2023 Dec 29;17(12):e0011858. doi: 10.1371/journal.pntd.0011858 (PMC10783723; doi:10.1371/journal.pntd.0011858)
Supplement: S2 Table — (DOCX) [file pntd.0011858.s004.docx]

**S2 Table: List of primers used for miRNA validation in the study**

| **S.No** | **Target Gene** | **Primer Sequence (5’-3’)** |
| --- | --- | --- |
| **1.** | **hsa-miR-19** | TGTGCAAATCCATGCAAAACTGA |
| **2.** | **hsa-miR-125a** | CGGCGTCCCTGAGACCCTTT |
| **3.** | **hsa-miR-146a** | GCGTGAGAACTGAATTCCA |
| **4.** | **hsa-miR-155** | GACTGTTAATGCTAATCGTGATAG |
