# Supplementary material for: Taenia solium excretory secretory proteins (ESPs) suppresses TLR4/AKT mediated ROS formation in human macrophages via hsa-miR-125
Source: PLoS Negl Trop Dis. 2023 Dec 29;17(12):e0011858. doi: 10.1371/journal.pntd.0011858 (PMC10783723; doi:10.1371/journal.pntd.0011858)
Supplement: Attachment — Submitted filename: PNTD-D-23-00633 (3) (2).pdf [file pntd.0011858.s005.pdf]

PLOS Neglected Tropical Diseases

Taenia solium excretory secretory proteins (ESPs) suppresses TLR4/AKT mediated ROS formation in human macrophages via hsa-miR-125

--Manuscript Draft--

**Manuscript Number:**	PNTD-D-23-00633
**Full Title:**	Taenia solium excretory secretory proteins (ESPs) suppresses TLR4/AKT mediated ROS formation in human macrophages via hsa-miR-125
**Short Title:**	Taenia solium ESPs effect on AKT
**Article Type:**	Research Article
**Keywords:**	Neurocysticercosis; Central nervous system; Excretory secretory proteins; Reactive oxygen species; Taenia solium
**Abstract:**	Background: Helminth infections are a menace affecting 24% of world population. They continue to increase global disease burden as their unclear pathology imposes serious challenges to patient management. Neurocysticercosis (NCC) is classified as neglected tropical disease and is caused by larvae of cestode Taenia solium. The larvae infect humans and localizes in central nervous system and causes NCC; a leading cause of acquired epilepsy in the developing world. The parasite has an intricate antigenic make-up and causes active immune suppression in the residing host. It communicates with host via its secretome which is complex mixture of proteins also called excretory secretory product (ESPs). Understanding the ESPs interaction with host can bring to light therapeutic intervention hot spots. In our research, we studied the effect of T. solium ESPs on human macrophages and investigated the post-translation switch involved in immunopathogenesis.<br>Methodology: T. solium cysts were cultured in vitro to get ESPs and used for treating the macrophages. These macrophages were studied for cellular signaling and miRNA expression and quantification at transcript level and protein level,<br>Conclusion: We found that T. solium ESPs treatment to human macrophages leads to activation of Th2 immune response. A complex cytokine expression by macrophages was also observed with both Th1 and Th2 cytokines in milieu. But, at the same time ESPs modulated the macrophage function by altering the host miRNA expression as seen with altered ROS activity, apoptosis and phagocytosis. This leads to activated yet compromised functional macrophages, that provides a niche to support parasite survival. Therefore T. solium secretome induces Th2 phenomenon in macrophages which may promote parasite's survival and delay their recognition by host immune system.
**Additional Information:**	
**Question**	**Response**
**Government Employee**<br><br>Are you or any of the contributing authors an employee of the United States government?<br><br>Manuscripts authored by one or more US Government employees are not copyrighted, but are licensed under a CC0 Public Domain Dedication, which allows unlimited distribution and reuse of the article for any lawful purpose. This is a legal requirement for US Government employees.	No - No authors are employees of the U.S. government.

Powered by Editorial Manager® and ProduXion Manager® from Aries Systems Corporation

This will be typeset if the manuscript is accepted for publication.	
**Financial Disclosure**<br><br>Enter a financial disclosure statement that describes the sources of funding for the work included in this submission. Review the submission guidelines for detailed requirements. View published research articles from *PLOS Neglected Tropical Diseases* for specific examples.<br><br>This statement is required for submission and **will appear in the published article** if the submission is accepted. Please make sure it is accurate.<br><br>**Funded studies**<br>Enter a statement with the following details:<br>• Initials of the authors who received each award<br>• Grant numbers awarded to each author<br>• The full name of each funder<br>• URL of each funder website<br>• Did the sponsors or funders play any role in the study design, data collection and analysis, decision to publish, or preparation of the manuscript?<br><br>Did you receive funding for this work?	Yes
Please add funding details.<br>as follow-up to "**Financial Disclosure**<br><br>Enter a financial disclosure statement that describes the sources of funding for the work included in this submission. Review the submission guidelines for detailed requirements. View published research articles from *PLOS Neglected Tropical Diseases* for specific examples.<br><br>This statement is required for submission and **will appear in the published article** if the submission is accepted. Please make sure it is accurate.	AP is supported through research grants BT/PR26841/MED/122/121/2017 Department of Biotechnology, Government of India, New Delhi and ECR/2016/000817/LS from SERB, New Delhi.

Powered by Editorial Manager® and ProduXion Manager® from Aries Systems Corporation

**Funded studies**<br>Enter a statement with the following details:<br>• Initials of the authors who received each award<br>• Grant numbers awarded to each author<br>• The full name of each funder<br>• URL of each funder website<br>• Did the sponsors or funders play any role in the study design, data collection and analysis, decision to publish, or preparation of the manuscript?<br><br>Did you receive funding for this work?"	
Please select the country of your main research funder (please select carefully as in some cases this is used in fee calculation).<br>as follow-up to "**Financial Disclosure**<br><br>Enter a financial disclosure statement that describes the sources of funding for the work included in this submission. Review the submission guidelines for detailed requirements. View published research articles from *PLOS Neglected Tropical Diseases* for specific examples.<br><br>This statement is required for submission and **will appear in the published article** if the submission is accepted. Please make sure it is accurate.<br><br>**Funded studies**<br>Enter a statement with the following details:<br>• Initials of the authors who received each award<br>• Grant numbers awarded to each author<br>• The full name of each funder<br>• URL of each funder website<br>• Did the sponsors or funders play any role in the study design, data collection and analysis, decision to publish, or preparation of the manuscript?<br><br>Did you receive funding for this work?"	INDIA - IN

**Competing Interests**<br><br>On behalf of all authors, disclose any competing interests that could be perceived to bias this work.<br><br>This statement will be typeset if the manuscript is accepted for publication.<br><br>Review the instructions link below and PLOS NTDs' competing interests policy to determine what information must be disclosed at submission.	None
**Data Availability**<br><br>Provide a **Data Availability Statement** in the box below. This statement should detail where the data used in this submission can be accessed. This statement will be typeset if the manuscript is accepted for publication.<br><br>Before publication, authors are required to make all data underlying their findings fully available, without restriction. Review our PLOS Data Policy page for detailed information on this policy. Instructions for writing your Data Availability statement can be accessed via the Instructions link below.	All relevant data used in this study are included in the manuscript and supplementary files or publicly available.

Manuscript

Click here to access/download;Manuscript;ROS manuscript main.docx

Title: *Taenia solium* excretory secretory proteins (ESPs) suppresses TLR4/AKT mediated ROS formation in human macrophages via hsa-miR-125

Naina Arora[1], Rimanpreet Kaur[1], Suraj Singh Rawat[1], Anand Kumar Keshri[1], Rajeev Kumar[2], Amit Mishra[3], Amit Prasad[1*]

[1]School of Biosciences and Bioengineering, Indian Institute of Technology Mandi, Mandi, Himachal Pradesh, India 175005

[2]CSIR-Institute for Himalayan Bioresource Technology, Palampur, Himachal Pradesh, India 176061

[3]Cellular and Molecular Neurobiology Unit, Indian Institute of Technology Jodhpur, Rajasthan, India 342011

*To whom correspondence should be addressed: Amit Prasad; E-mail: amitprasad@iitmandi.ac.in

Abstract:

Background: Helminth infections are a menace affecting 24% of world population. They continue to increase global disease burden as their unclear pathology imposes serious challenges to patient management. Neurocysticercosis (NCC) is classified as neglected tropical disease and is caused by larvae of cestode *Taenia solium*. The larvae infect humans and localizes in central nervous system and causes NCC; a leading cause of acquired epilepsy in the developing world. The parasite has an intricate antigenic make-up and causes active immune suppression in the residing host. It communicates with host via its secretome which is complex mixture of proteins also called excretory secretory product (ESPs). Understanding the ESPs interaction with host can bring to light therapeutic intervention hot spots. In our research, we studied the effect of *T. solium* ESPs on human macrophages and investigated the post-translation switch involved in immunopathogenesis.

Methodology: *T. solium* cysts were cultured *in vitro* to get ESPs and used for treating the macrophages. These macrophages were studied for cellular signaling and miRNA expression and quantification at transcript level and protein level,

Conclusion: We found that *T. solium* ESPs treatment to human macrophages leads to activation of Th2 immune response. A complex cytokine expression by macrophages was also observed with both Th1 and Th2 cytokines in milieu. But, at the same time ESPs modulated the macrophage function by altering the host miRNA expression as seen with altered ROS activity, apoptosis and phagocytosis. This leads to activated yet compromised functional macrophages, that provides a niche to support parasite survival. Therefore *T. solium* secretome induces Th2 phenomenon in macrophages which may promote parasite's survival and delay their recognition by host immune system.

Keywords: Neurocysticercosis; Central nervous system; Excretory secretory proteins; Reactive oxygen species; *Taenia solium*

Author Summary

This article discusses the research conducted on the effect of *Taenia solium* excretory secretory proteins (ESPs) on human macrophages and its role in the immunopathogenesis of Neurocysticercosis (NCC). NCC is a neglected tropical disease caused by larvae of *T. solium*, which infects humans and localizes in the central nervous system. The larvae cause NCC, a leading cause of acquired epilepsy in the developing world. The study found that *T. solium* ESPs induce a Th2 immune response in macrophages, which may promote parasite survival and delay their recognition by the host immune system. The research further showed that ESPs modulate macrophage function by altering host miRNA expression, leading to activated yet compromised functional macrophages that provide a niche for parasite survival. The study provides insights into the interaction of ESPs with the host, revealing therapeutic intervention hot spots to manage NCC and other helminthic infections. In the absence of suitable animal model for NCC, our study elucidates important and novel finding of parasite mediated suppression of host immune system.

1. Introduction:

More than a quarter of the population in the world's population is threatened by helminthic infections, which cause a significant disease burden and associated disabilities. Chronic helminthic infections skew the immune response towards T-helper 2 (Th2) by altering the early immune responses generated by macrophages and dendritic cells [1].

Macrophages are front line warriors of immune system and play a crucial role in defining the immune response as they influence several biological processes due to their plasticity [2]. Macrophages are a very versatile class of immune cells that can adapt to and differentiate into different subsets depending on the environmental stimuli. These heterogeneous immune cells have been described for their different activation states: "classically activated" M1 macrophages, "alternatively activated" M2 & M2 subtypes macrophages, regulatory macrophages and less defined subsets like tumor associated macrophages [3,4]. Since macrophages encounter and recognize a wide range of insults classified as "Danger Associated Molecular Patterns" (DAMPs) & "Pathogen Associated Molecular Patterns" (PAMPs) via exogenous or endogenous "Pathogen Recognition Receptors" (PRRs) for example Toll like receptors (TLRs), they can exhibit protective or pathogenic roles in different stages of an infection or a disease. The M2 macrophages known as "alternatively activated macrophages" are induced by Th2 cytokines like IL-4 and are required for tissue repair, wound healing, unregulated inflammatory and autoimmune response [5]. The role of helminths derived products in determining the M2 polarization has been reported [6] but has not been so far fully defined, especially for cestode "*Taenia solium*". Taenia is a highly prevalent species of helminth infecting humans and swine [7]. The larvae infiltration to human

central nervous system (CNS)/brain causes severe infection i.e., Neurocysticercosis (NCC), which causes epilepsy. Understanding the ESPs interaction with the host may reveal therapeutic intervention hot spots to manage NCC and other helminthic infections.

NCC is an extremely widespread CNS associated infection and is the single most common cause of late onset of acquired epilepsy caused by larvae of *T. solium*, more frequently in developing tropical countries [8-10]. As per World Health Organization (WHO) report (https://www.who.int/news-room/fact-sheets/detail/taeniasis-cysticercosis 2015), out of 50 million new epileptic incidences reported worldwide, approximately one third cases are due to NCC in endemic regions [11]. The WHO Food Borne Disease Burden Epidemiology Reference Group 2015 had identified "*T. solium* as a leading cause of deaths by food borne disease considerable to 2.8 million disability adjusted life years loss (DALYs)" (https://www.who.int/news-room/fact-sheets/detail/taeniasis-cysticercosis 2015.)[11]. Although the endemicity of the disease is restricted to sub-tropical and tropical parts of low- and middle-income countries, but incidences from developed countries are also frequently reported owing to frequent travel and immigration of individuals from endemic region to non-endemic areas [12]. The clinical presentation of NCC patients is pleomorphic. The most common clinical presentation is recurrent epileptic seizures [13-15]. To date, the immune pathogenesis of this disease is unclear, due to complex biology of the parasite that includes the lack of knowledge about the role of different antigenic pools generated by this parasite, especially the parasite secreted antigens [15-17].

Unlike intra-cellular parasites which resides inside the host cells, *T. solium* is an extracellular parasite and parasite's secreted factors or secretome are predominantly

involved in shaping up of host immunity [18-20]. The parasite secretory factors or excretory secretory proteins (ESPs) are the parasite's mode of communication with the host, and they interfere with host immune-signaling mechanisms and immune homeostasis. These ESPs constantly keep changing their composition as per the environmental stimuli received by the parasite to support its survival. The ESPs of *T. solium* are a complex mixture of proteins as they are constantly secreted by live metacestodes hence identifying them is a daunting task. Fortunately, the whole genome of *T. solium* had been published [21] and the WGA for secretome had identified 838 proteins as ESPs. Out of these 838 proteins, mRNA for 347 of them was identified and the KEGG analysis had identified 166 pathways that includes protein processing, pathways in cancer, focal adhesion, PI3K-Akt pathway, Wnt signaling, glycerophospholipid metabolism etc. [22]. These reports had helped us in our previous studies where we had identified the different proteins present in *T. solium* secretome and kinome associated with different cellular processes like cell proliferation, cell adhesion, migration, and maturation [23-24]. This also established ESPs as a significant source of "immunogenic proteins" due to their availability to be processed and recognized by the host immune system. These findings had made the ESPs proteins more lucrative for further investigation. Effect of various antigens like vesicular fluid, cyst wall or crude lysate of *T. solium* on immune cells have been reported [25]. However, the effect of ESPs antigens on macrophages was never explored before, though such studies are essential for our understanding of helminth-host interaction. Hence, in this study we investigated the immune response of *T. solium* ESPs on human macrophages.

2. Material and methods:

2.1 Isolation of excretory secretory proteins (ESPs)

The ESPs were made as previously described [23]. Briefly, cysts were isolated from naturally infected swine after sacrifice at local butcher house and washed thrice thoroughly in chilled PBS supplemented with antibiotics. Cysts were cultured in RPMI 1640 only and RPMI1640 supplemented with 10% FBS for 24hrs. After 24hrs, culture supernatant was collected and filtered through 0.45uM filtered and this was considered as Excretory secretory proteins (ESPs) and stored at -80°C for further experiments.

2.2. Cell culture and treatment

The human origin cell lines U937 and THP-1 were purchased from the national cell repository at National Center for Cell Sciences (NCC), Pune, India. Cells were cultured as per guideline in RPMI 1640 culture medium supplemented with 10% FCS. The cells were differentiated to macrophages and treated with 20μg/ml of ESPs antigens for 24 hrs for all our experiments, until stated otherwise.

2.3. Extraction of RNA and QPCR

The cultured cells were lysed with TRIzol™ LS reagent for total RNA extraction and was stored at -80°C till use. All the procedures were performed following protocols as described previously [26]. RNA was reverse transcribed with a commercial kit (iScript, BioRad) to generate initial cDNA species and the samples were stored at -20ºC until use. The list and sequence of primers used for QPCR of cytokines, TLRs and β-actin gene and annealing details used are given in **supplement table 1**.

2.4. Cytometric bead assay for cytokines

Cytokine quantification at protein level was also done using cell bead array technology with BD "CBA Human Th1/Th2/Th17 Cytokine Kit" (Cat No.560484) as per

manufacturer's guideline. The data was acquired on BD LSR Fortessa and analysis was done using BD CellQuest software.

2.5. Western blot for TLRs

Expression of TLRs was further confirmed at translation level by doing WB. Primary monoclonal antibodies (β-actin #8457, TLR4 #12276 AKT #4691 pAKT#4056) were purchased from Cell Signaling (USA) and used as per manufacturer instruction. The blot was developed by the ECL method.

2.6. ROS measurement:

2.6.1. By Cytochrome C assay: To assess the superoxide production from macrophages, SOD-inhibitable cytochrome-c assay was performed. In brief, $0.5x10^6$ cells were pre-treated with antigen and incubated with 1.5 mg/ml of cytochrome c (from equine heart; Sigma) for 5 minutes at room temperature with or without 100 U/mL of SOD. The reduction in cytochrome c was recorded for each sample by centrifuging cells and measuring the absorbance of the supernatant at 550 nm using a spectrophotometer. The difference between absorbance in samples with and without SOD was recorded.

2.6.2. By Flow cytometer: The ROS generation was quantified through flow cytometer using CellROX orange flow cytometry assay kit. In brief, $5X10^5$ cells were taken and incubated with and without ESP for the mentioned time. To prepare negative and positive control, cells were treated with N-acetylcysteine (NAC) (1mM) for one hour to increase the antioxidant capability of the cell. For positive control tert-butyl hydroperoxide (TBHP) (200uM) was added to cells and incubated for 30-60 min. TBHP was also added to negative control post incubation with NAC. Once the controls were set up. The cells for treatment group and controls were stained with CellROX

reagent (250uM) and incubated for 30-60 min at 37ºC, following staining cells were acquired and analyzed on 532nm channel.

2.7. Phagocytosis assay

Zymosan BioParticles (Invitrogen, Carlsbad, CA) were incubated with 10% human serum in PBS for 1 hour to opsonize. The differentiated THP-1 cells were suspended in HBSS at a density of $1x10^7$/ml. The opsonized Zymosan particles and cells were then incubated at a 1:50 ratio at either 37ºC or 4ºC with end-to-end rotation for 1 hour. "Extracellular fluorescence was quenched by adding trypan blue, and the phagocytosis index (PI) was calculated as the number of bioparticles engulfed by 100 macrophages" as described previously [27]. For each group >200 cells were counted.

2.8. In vitro bacterial killing assay

Escherichia coli (strain 19138; ATCC, Manassas, VA) and Staphylococcus aureus (strain 10390; ATCC, Manassas, VA) were freshly cultured overnigh t and resuspended in PBS at an OD600 of 0.20. They were then opsonized with 10% human serum for 90 minutes at 37ºC in a water bath. The cells & bacteria were incubated together at a ratio of 1:10 for E. coli and 1:20 for S. aureus for different time intervals of 0, 30, 60, and 120 minutes at 37ºC with intermittent shaking. To lyse the cells, autoclaved H_2O was added, and diluted aliquots were spread on LB agar (E. coli) or Blood agar (S. aureus) plates. The plates were then incubated overnight at 37ºC, and the colony-forming units (CFU) were counted. A bacterial suspension without any cells was used as an input control.

2.9. Extraction of miRNA and microarray

Total miRNA was extracted from cultured cells by miRNA easy mini kit (Cat No./ID: 217004), Qiagen, USA as per the manufacturer's protocol and was stored at -80°C till use. In brief, the cells were lysed using lysis buffer (500ul) and incubated at RT for 5 min, followed by addition of chloroform (140ul), incubated at RT for 2-3 mins and centrifuged at 12000g for 15 mins, 4°C. Aqueous phase was taken to new tube and mixed with 1.5vol of 100% ethanol. Resulting mix was transferred to the spin column and centrifuged at 12000g for 15s. Flow through was discarded, spin column was washed with RWT once (700ul) followed by RPE (500μl) twice for 15s and 1min and RNA was eluted in 11ul of RNAse free water. The RNA obtained was quantified and checked for RIN value before proceeding to microarray (RIN >9).

For miRNA expression, a microarray experiment was performed on GeneChip™ miRNA 4.0 Array (Cat No. 902412). Input miRNA taken was 400ng and the poly(A) tailing, flash tag labeling and hybridization cocktail were done as described in manufacturer's protocol for FlashTag Biotin HSR RNA labeling kit (Affymetrix 901910). Sample to chip hybridization was done overnight, the hybridization cocktail was removed, and the array was stored in array holding buffer, followed by washing and staining the array using fluidics console after which the array was loaded into the array scanner.

For miR relative expression, the extracted miR was reverse transcribed using miScript II RT Kit, Qiagen (#218161) using HiSpecBuffer (present in the kit). The cDNA was used to study relative expression using Qiagen miScript SYBR Green PCR Kit (#218075) that comes with universal primer. Primer sequences for miR are given in supplementary. RNU6 Qiagen primer assay, miScript Primer Assay (#218300) was used for endogenous control.

2.10. miRNA Transfection

mirVANA miRNA mimics were ordered from Ambion ® Thermo fisher (hsa-miR-1246 **ID:**MC13182, hsa-miR-6820-5p **ID:** MC26881, hsa-miR-3201 **ID:** MC16523, hsa-miR-125a-5p **ID:** MC12561). The 5 nmol lyophilized miRNA mimic was resuspended in nuclease free water to 10uM working stock. We transfected $1x10^6$ cells with 30pmol of miRNA mimic using Neon™ Transfection system as described earlier [28] In brief the cell and miRNA mimic mixture were loaded in the Neon™ transfection tip and electroporated at 500 volts with five pulses for 10ms according to manufacturer’s protocol. The cell and miRNA mixture were then plated and incubated for min 6 hours prior to treatment (for cell attachment) at 37°C and 5% CO2, post incubation the cells were washed with PBS to remove dead cells and stimulated with ESP/LPS as per the test group and further incubated for 24 hours. Post treatment, cells were washed and scrapped as pelleted down for western blotting and RNA extraction followed by QPCR as explained above. The list of primers used for validation study is given in **supplement table 2**.

2.11. Enzyme linked immunosorbent assay (ELISA)

ELISA samples were prepared as described previously (Singh et al., 2015) Cell Culture soup was collected, aliquoted and stored at -80° C for further use. After estimating protein concentrations all the samples were diluted to adjust the final protein concentration of 500 µg/ml and equal amounts of proteins were subjected to ELISA using commercially available kits (Invitrogen, USA) as per manufacturer’s instructions. The results were expressed as picograms of cytokine/mg (pg/mg), based on the standard curve sketched with standard provided with the kits. Similarly, serum samples were quantified for different cytokines.

2.12. Statistical analysis

The data here presented as “mean ± SD” from at least three independent experiments with three technical replicates each, if specified otherwise.

One way ANOVA F test was applied to analyze variations in cytokine concentrations among cell groups stimulated with ESPs, LPS and treatment control. The Comparison for two groups with three different stimulations was made employing “multiple comparisons using Bonferroni “T- test”. Correlations were calculated using “Pearson’s test”. A “P value” of less than or equal to 0.05 was considered statistically significant. The comparisons of averages between groups were analyzed by “one way ANOVA”, and the “Dunn Multiple Comparison Test” was further used to determine significant differences between groups at the significance level of $P < 0.05$ (* $P < 0.05$ and ** $P < 0.01$) [29].

2.13. Ethics statement

All the work was performed after taking required approval from the Institute Ethics Committee and Institute Biosafety Committee of IIT Mandi.

3. Results

3.1. T. solium ESPs give both Th1/Th2 cytokines

Several helminth derived products had been found to be immunogenic and inducing Th2 immune response and hence we also looked for the effect of ESPs on macrophages. The Th1/Th2 cytokines were measured at transcription (by qPCR) and translation level (by Cytometric bead assay). We found significantly elevated levels of

Th1 cytokines like TNF-α, IL-1β & IL-6 along with signature Th2 cytokines IL-4 & IL-10 by qPCR (Figure 1 A). The same mixed cytokines response was observed at protein level also with TNF-α, IL-1β and IL-6 along with signature Th2 cytokines IL-4 and IL-10 significantly high in ESPs treated macrophages (Figure 2). The progression of cytokines spectrum is given in **Supplement fig 5**, showing peak for TNF-α, IL10 and IL-6 at 12 hrs post stimulation.

A.)

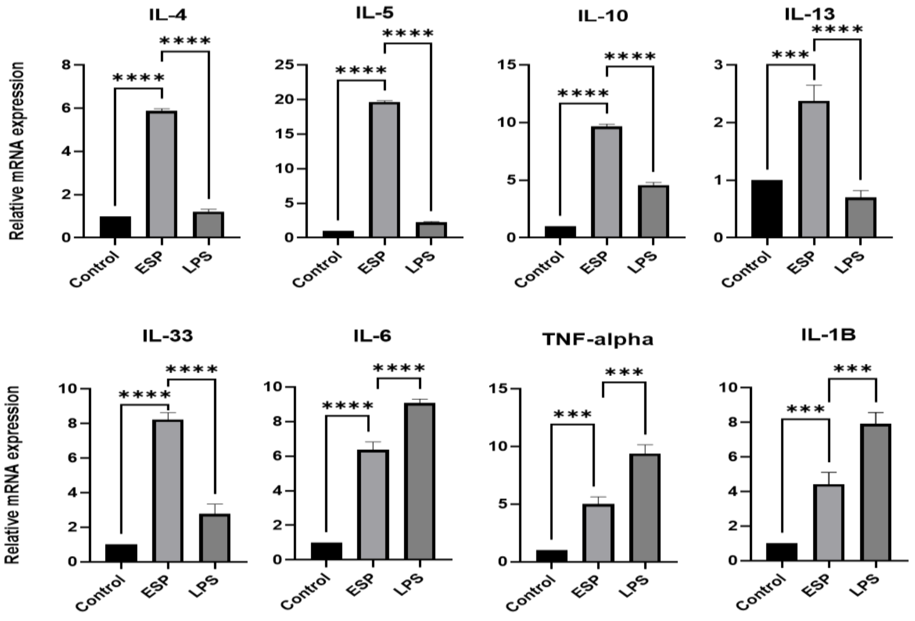

B.)

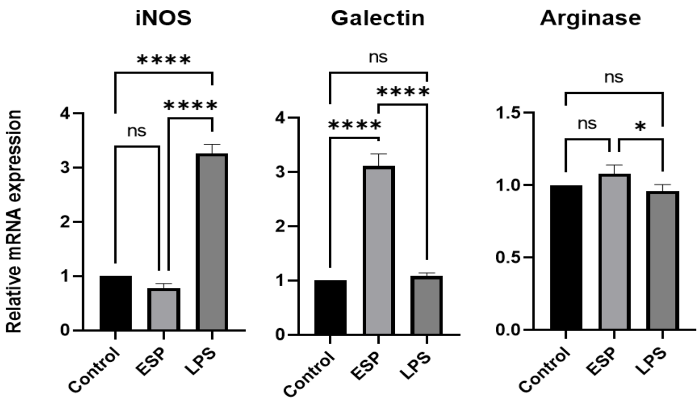

Figure 1: Relative gene expression of Th1 and Th2 cytokines (A.) and cell surface markers (B.) in *Taenia solium* ESPs treated THP-1 derived macrophages by qPCR. The *T. solium* ESPs stimulated macrophages had shown both Th1 and Th2 cytokines expression.

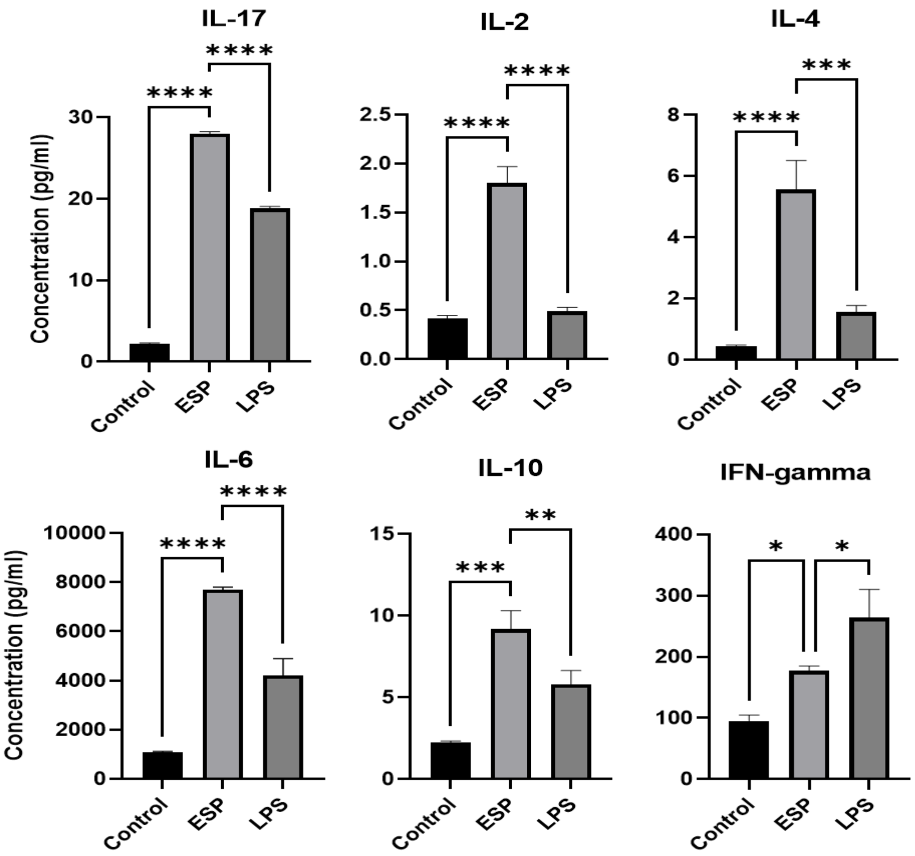

Figure 2: Absolute quantification of secreted cytokines in the milieu of *Taenia solium* ESPs treated THP-1 derived macrophages measured using cytometric bead assay.

3.2. ESPs induce M2 polarization of macrophages

Helminth-derived products play important role in deciding the fate of monocytes and it is reported that they promote alternatively activated macrophage (AAM) lineage [6,30]. The cells treated with ESPs were evaluated for M1/M2 macrophage lineage marker and we found significantly high expression of Galectin 3

compared to control (**Figure 1 B**). We also observed higher expression of Arginase, though this was not significant (p=0.063) (**Figure1 B**).

3.3. T. solium ESPs effect on TLRs

The TLRs play important role as the upstream signaling molecules of inflammation and several of them has been associated with NCC pathogenesis. In a murine model study of NCC, all the TLRs (from 1-9) were found to be abundant in microglial cells except the TLR 5 [31]. But the expression of the TLRs induced by ESPs antigens in macrophages has not been yet reported. After stimulation of macrophages with ESPs, we also observed significantly high expression of genes for all the TLRs (1-9) except for the TLRs 7, **Figure 3**.

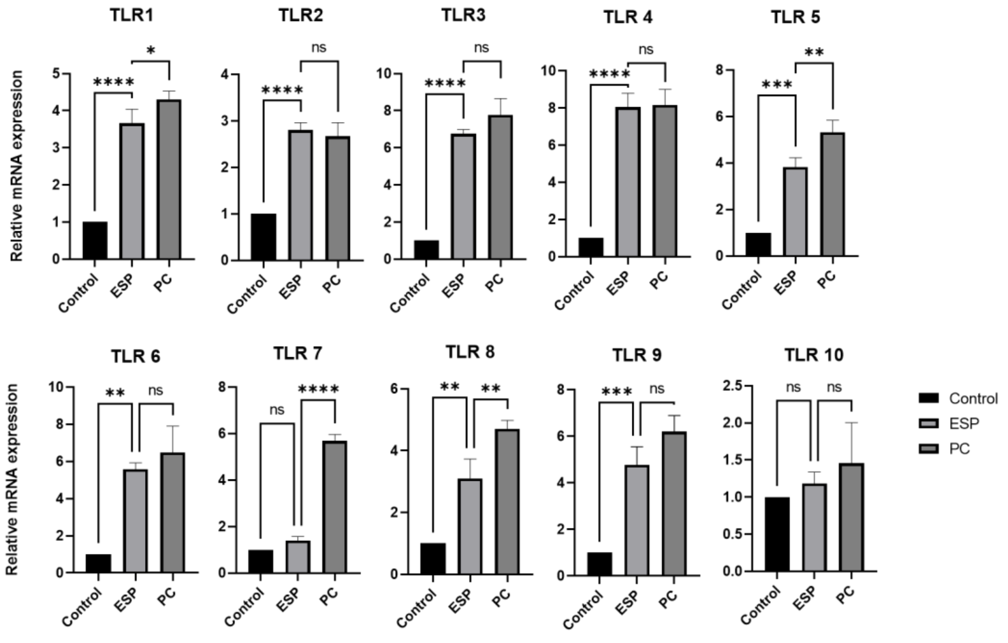

Figure 3: Relative expression of TLRs by ESPs treated macrophages estimated by QPCR. PC- positive control, TLR agonists used for each of the TLRs from Human TLR agonist kit -InVivoGen Cat Code # tlrl-kit1hw (TLR1/TLR2 Agonist: Pam3CSK4, TLR2 Agonist: HKLM, TLR2/TLR6 Agonist: FSL-1,TLR3 Agonist: Poly(I:C)-HMW,

TLR3 Agonist: Poly(I:C)-LMW, TLR4 Agonist: LPS-EK standard (LPS E.coli K12) , TLR5 Agonist: FLA-ST standard (Flagellin S. typhimurium), TLR7 Agonist: Imiquimod,TLR8 Agonist: ssRNA40/LyoVec™, TLR9 Agonist: ODN2006)

The diverse make-up of ESPs was rich in ligands for all the TLRs. We specially checked the expression of TLR4 by doing western blot too as it has been reported that TLR4 is directly related with symptomatic NCC [32] and have been associated with immune pathogenesis of NCC. And to our surprise, we noticed no change in TLR4 expression at protein level (**Figure 4**) though we had observed higher expression of TLR4 genes by qPCR (**Figure 3**).

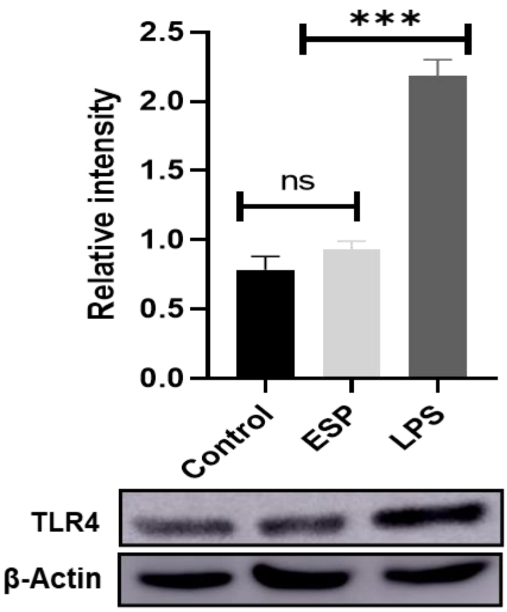

Figure 4: Western blot analysis of TLR4 expression in macrophages treated with *Taenia solium* ESPs antigens. The densitometry analysis of WB bands was normalized to housekeeping protein β-Actin. The results shown are representative of three independent experiments.

3.4. Reduced Akt activity, ROS production and bacterial killing capability

TLR4 has been found to be directly involved in the activation of master regulatory molecule Akt by converting to pAkt, and pAkt is responsible for generation of reactive oxygen species (ROS) [33-34]. The ROS are indispensable mechanism of the innate immune response against infections and immensely contribute to bacterial killing by an immune cell [27]. To establish the effect of ESPs on TLR4/pAkt mediated ROS production, we measured pAkt/Akt by western blot and ROS production by Cytochrome C/DFCDA method (2′,7′-Dichlorofluorescin diacetate; CellROX ROS orange reagent, Invitrogen, USA). We found reduced pAKT in macrophages incubated with ESPs compared to LPS or negligible increase compared to control, when stimulated with fMLP (**Figure 5A**). We checked the Akt activation in biological settings when they were kept for phagocytosis of *E. coli* (**Figure 5B**) and noticed reduced pAkt. This suggested that ESPs treated macrophages might have defects in their bacterial killing capability and cell survival. Hence, we evaluated these treated macrophages for their bacterial killing capability following previously published protocol [27]. In brief, cells were incubated with Gram negative (*E. coli*) & Gram positive (*S. aureus*) bacteria for 120 minutes, cells were washed and lysed after incubation, then lysate was plated on agar plate. The plates were left overnight at 37°C. We observed significantly ($p<0.001$) more colony-forming units (CFU) in ESPs treated cells (163) when compared to control (102) (**Figure 5C**). As macrophages generate ROS to kill bacteria, so next we evaluated the effect of ESPs on ROS production on these cells. We performed cytochrome-c reduction assay, this assay measures the total amount of ROS produced by a cell, and we noticed significantly less ROS production by ESPs treated cells (**Figure 6**). To confirm our observation that this reduced ROS is due to less Akt activity, we used small molecule SC79 (Merckmilipore, CAS 305834-

79-1; [34], an Akt activator along with ESPs and we saw recovery of phenotype. The macrophages treated with ESPs and SC79 had tentatively equal number of CFUs. This restoration of bacterial killing capability by Akt activator SC79 confirms the role of ESPs in decreasing Akt activity.

This diminished bacterial killing capability could stem from decreased phagocytic capability too as pAkt is also responsible for making f-Actin [35]. To assess this, we counted the number of zymosan-bioparticles engulfed by individual macrophages via an in-vitro phagocytosis assay (**Figure 5 D**). On average, we found 53 “serum-opsonized fluorescein-conjugated zymosan particles” were engulfed by 100 control macrophages (phagocytic index) (**Figure 5 D**). While in ESPs treated macrophages the number was significantly low i.e., 24 ($p<0.001$), suggesting a significant loss in phagocytic capability. Akt activation has been associated with cell survival also as they give survival signal [27], hence we evaluated the ESPs treated macrophage for apoptosis however we could not find any difference (**supplementary fig 6**).

A.) B.)

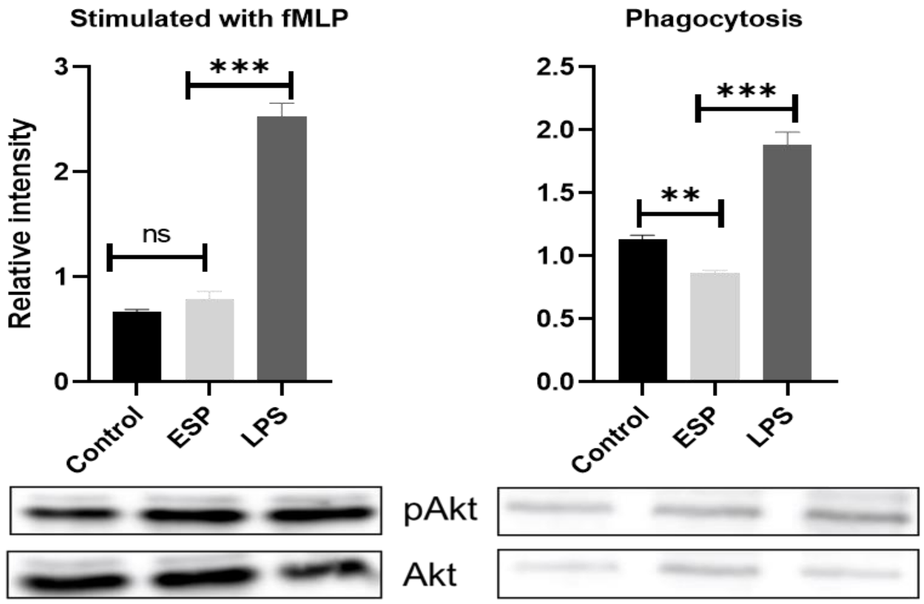

C.)

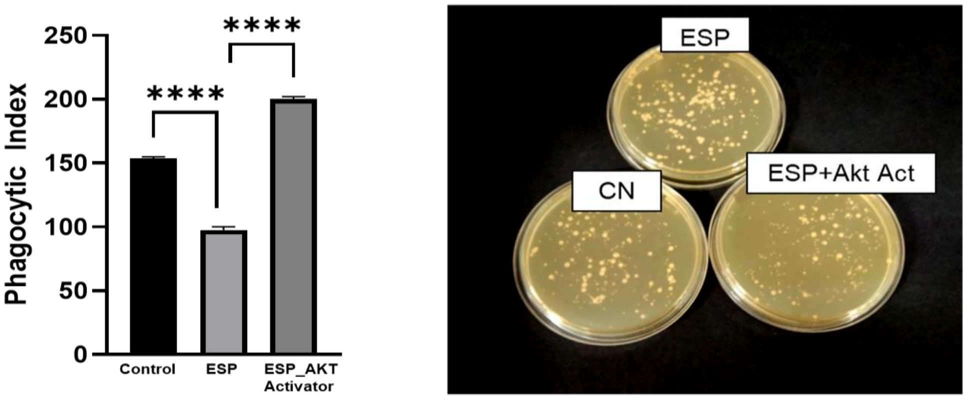

D.)

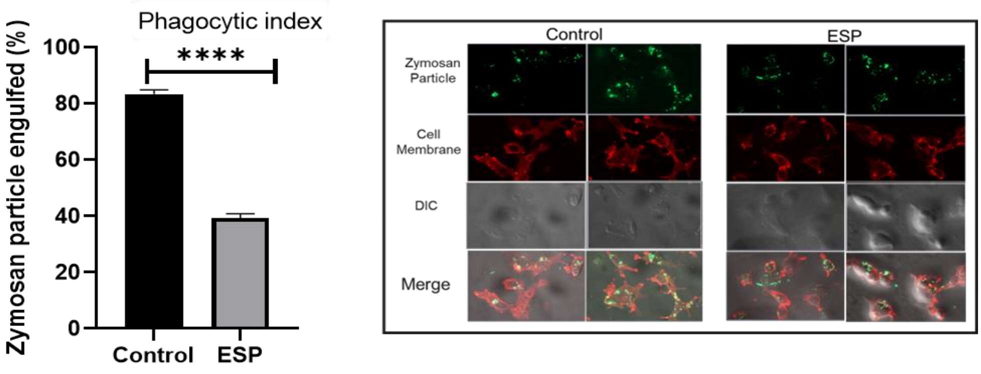

Figure 5: *Taenia solium* derived ESPs treatment suppresses Akt activation in THP-1 macrophages when stimulated with **A**.) fMLP or during B.) bacterial phagocytosis. C.) *Taenia Solium* ESPs treated macrophages had reduced bacterial (*E. coli*) killing capacity. D.)Macrophages treated with ESPs had less Zymosan bioparticles uptake, suggesting a decrease in phagocytotic capability. Flouresence microscopy (right). **B**.) Phagocytic index (left). The figure is representative of three independent biological

experiments, in five randomly selected high power view per slide.

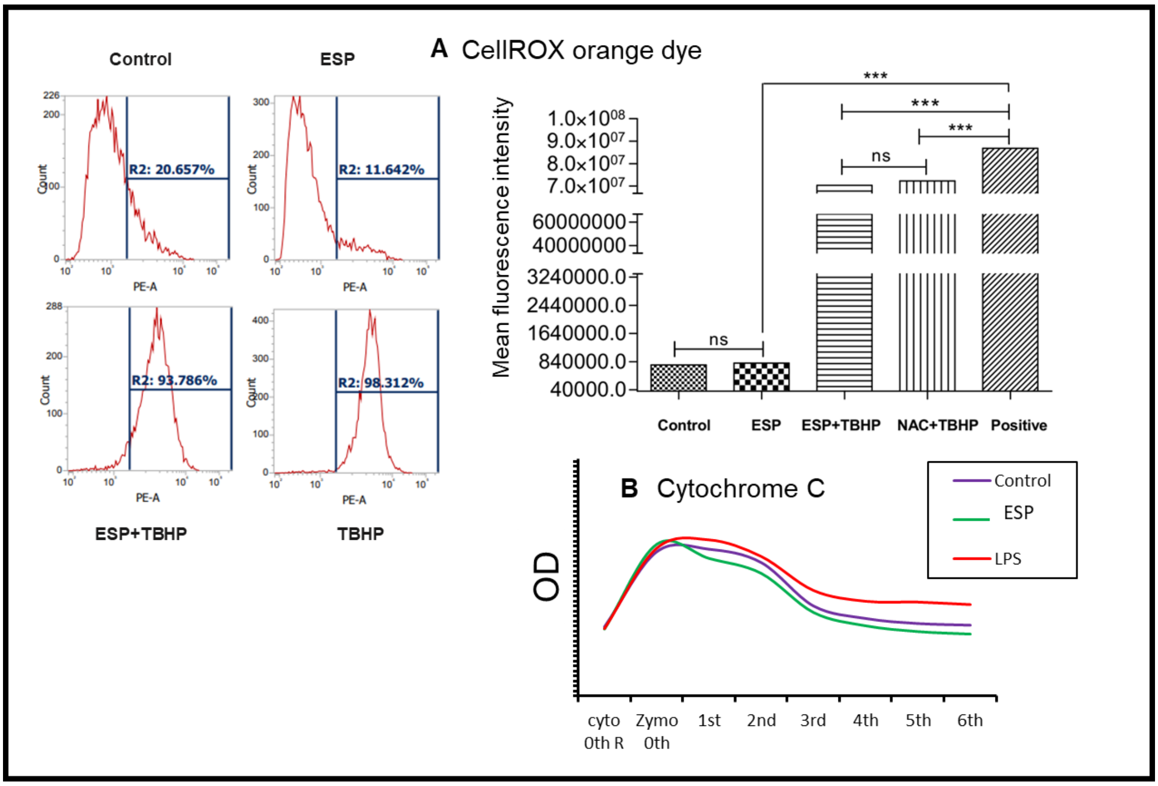

Figure 6: *Taenia solium* ESPs generate significantly less ROS in macrophages. **A**. THP-1 cells treated with ESPs+TBHP showed significantly less intenstiy when compared to cells treated with TBHP alone (positive control) in a flow cytometer based ROS assay **B**. The measurment of total ROS generation by Cytochrome C assay also confirmed reduced ROS in ESP treated macropahges.

3.5. Epigenetic control of TLR4 expression

Small non-coding RNAs (miRNA, siRNA and piRNA) are crucial regulators of gene expression and often execute their function by silencing target genes in a broad range of living forms including metazoans, fungi and plants [36-37]. The “miRs” or “miRNAs” belong to class of small non-coding RNAs. They regulate gene expression of multiple gene targets within the same or distinct signaling pathways by directly binding to target based on sequence complementarity at 3′ untranslated region (3′ UTR)

of target mRNA [38]. The role of miRNAs in helminthic infection has also been highlighted previously [39]. To identify the miRs related with TLR4/Akt/inflammation axis we did micro-RNA microarray of ESPs treated THP-1 macrophages. The pixel intensity obtained was normalized and converted to .chp file which was analyzed using Affymetrix transcriptome analysis console. The microarray raw data is available at https://www.ncbi.nlm.nih.gov/geo/query/acc.cgi?acc=GSE232005 (GSE232005) The heat map of miR microarray is given in **Figure 7 A**

A.)

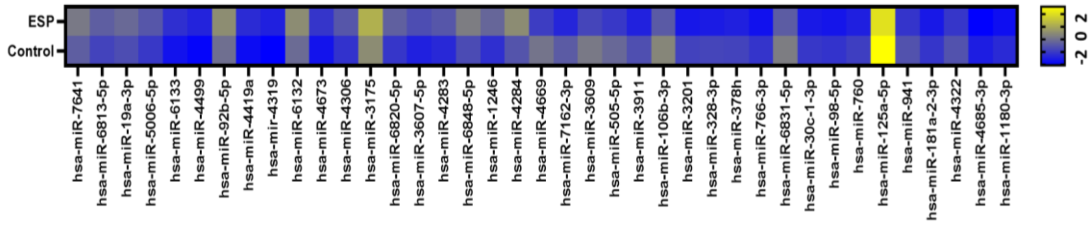

B.)

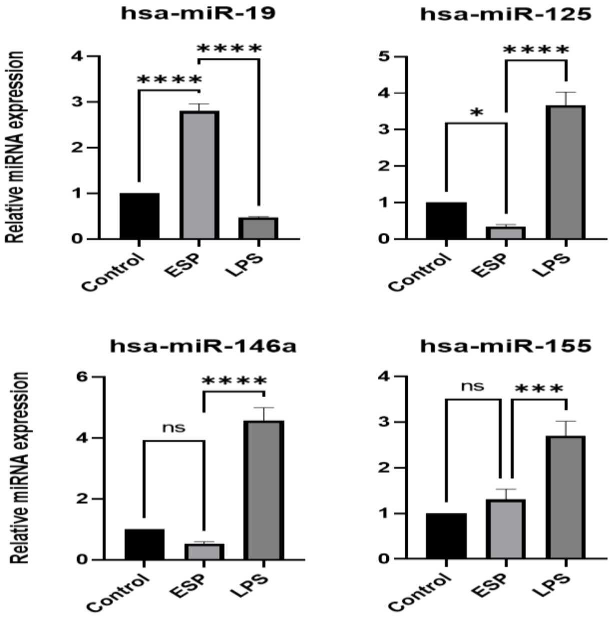

C.)

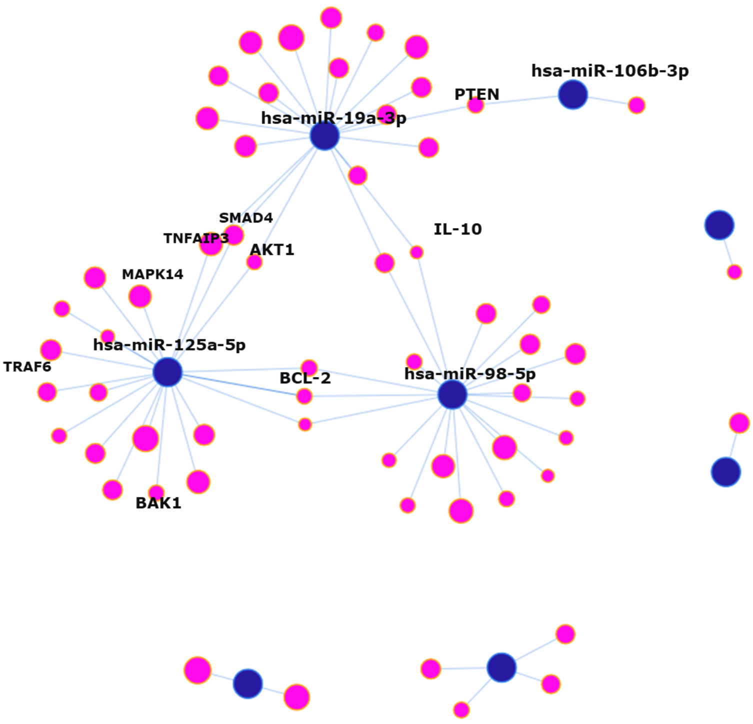

D.)

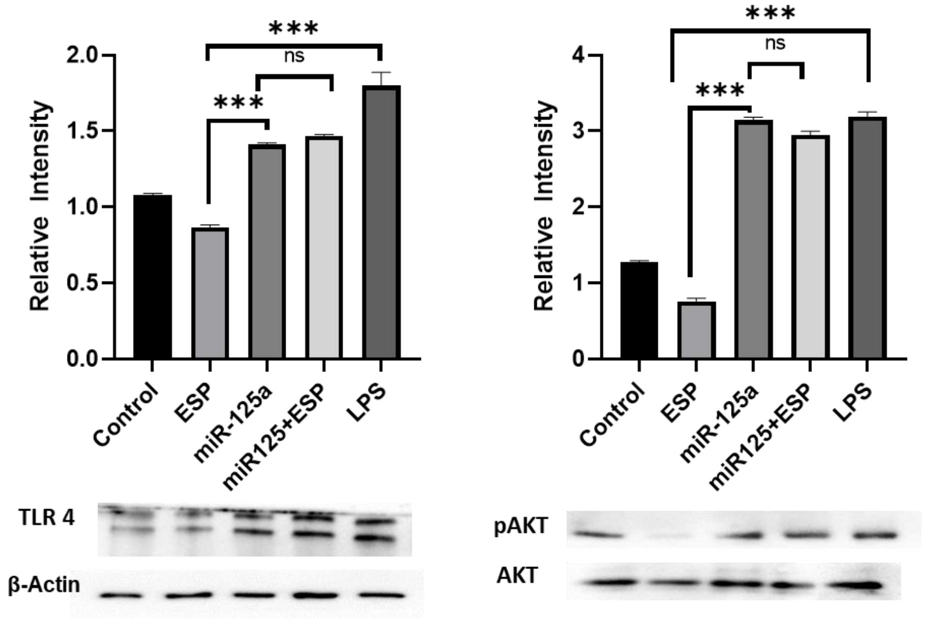

Figure 7. A. miRNA microarray of ESPs treated macrophages showed 57 differentially expressed miRNA, **B**. validation of microarray targets miRs by qPCR. **C.** Strongest miRNA interaction from microarray data map (http://userver.bio.uniroma1.it/apps/mienturnet/) D. Restoration of ESP treated macrophage function on transfection with has-miR-125.

From this list we selected miRs, whose expression had changed at least 1.5 folds and were searched in “Target scan” and “miRNet” for targets involved in TLR4/Akt/inflammation and further validated by qPCR. The miRNA interaction pathway was generated at MIENTURNET [40] (**Supplementary table 2**). The qPCR of selected miRs validated our microarray data and we observed significantly high expression ($p<0.005$) of hsa-miR-19 and down regulation of hsa-miR-125a, hsa-miR-146a and hsa-miR155 in ESPs treated differentiated THP-1 cells. It has been reported that has-miR-19 regulates Akt activity indirectly by regulating Phosphatase and tensin homolog (PTEN) enzyme, it positively regulates the activity of PTEN and high PTEN activity leads to less Akt activity [41]. We also noticed less pAkt in ESPs treated THP-1 macrophages. In contrast to upregulated miR-19, we observe downregulated miR-125 in microarray which is involved in Akt mediated apoptosis pathway. The expression of miRs in microarray strongly resonates with the finding of our study and establishes that *T. solium* ESPs immune modulates at the post transcriptional level and impairs the macrophage’s function. In myeloid cells has-miR-146 controls the expression of NF-κB, that in turn regulates NF-κB activation through a negative feedback loop [42] thus restricting pro-inflammatory cytokines expression. But we noticed down regulation of hsa-miR-146, hsa-miR-155 which correlated to diminished inflammation, supported by downregulated Akt pathway. To further validate the role of miRNA mediated immune suppression by *T. solium* ESPs, has-miR-125 was transfected in ESPs stimulated cells to see if it restores the phenotype. The ESPs primed macrophages were transfected with hsa-miR-125 since the downregulation of Akt pathway resonated with downregulation of hsa-miR-125 (**Figure 7C**), impairing the macrophage function and inflammation which is essential for elimination of larvae in

initial stage of infection [43]. In our experiments, we observed that ESPs primed macrophages transfected with has-miR-125 restored the TLR2/4 expression as compared to positive control. These miRs are involved in an array of functions from cell differentiation to maturation. The study suggests an immune modulatory effect of *T. solium* ESPs on these miRs and that sets up an environment to support parasite survival by disarming the inflammatory immune response. In another perspective, the dampened inflammation might be a tissue homeostasis mechanism required by the host to survive the parasite insult.

4. Discussion

On exposure to pathogens or other stimuli, macrophages respond by triggering inflammatory responses. This response is initiated by signaling cascades that are activated downstream of TLR & cytokine receptors. Once activated, the macrophages undergo transcriptional and epigenetic changes that lead to the production of pro-inflammatory cytokines and chemokines. The macrophages then migrate to the site of infection and eliminate the insult. Later, macrophages play a role in resolving the inflammation and preventing persistent inflammatory reactions that could damage surrounding tissues. Helminth derived antigens or the whole parasite can counteract pro-inflammatory responses generated during its infection and these antigens usually make immune response biased to Th2 type but also give some Th1 cytokines thus giving mixed cytokines profile [5-6,25]. This primarily Th2 type response assist host in parasite expulsion, tissue repair & regeneration, regulation of any inflammatory & autoimmune response. Some studies had been done involving *T. crassiceps* derived ESPs to identify the specific proteins [44-45] but this species infects rodents not human/swine and no study so far had been taken to understand their role in host immune

modulation. Spolski et al (2002) looked for the immuno-modulating capability of *T. crassiceps* ES and noticed ES from early larvae but not late harvested larvae could suppress T cell proliferation *in vitro* and a decline in the production of IFN-γ & IL-4 [45]. Terrazas et al. 2013 had reported defect in DC maturation, impaired Th1 cytokines production, and sensitivity towards TLR2 response that leads to Th2 polarization [46]. They had also reported more alternatively activated macrophages in mice model. Similarly, Chauhan et al (2014) also noticed reduced macrophages activation and Th2 polarization of myeloid cells when treated with soluble parasite ligands [47]. We also noticed both Th1 and Th2 cytokines elevated expression (IL-1β, TNF-α, IL-6, IL-4 & IL-10) along with expression of M2 macrophages markers. This shift of macrophage polarization towards M2 phenotype is essential to protect tissue damage from prolonged inflammation as M1 is directed to kill while M2 macrophage are directed to heal [48].

TLRs are encoded in the germline and act as pattern recognition receptors, they belong to innate immune system. Their primary role is to recognize a wide range of microbial products, and they are expressed on both immune and non-immune cells. TLRs are crucial for the body's defense against pathogens [49]. In an earlier study on murine cysticercosis model, all the TLRs except the TLR 5 was identified [31]. In the same model they found high expression of TLR 11-13 also, with TLR13 being the most expressive in all the cells and brain areas [50]. The TLR4 is one of the most studied receptors as it activates its downstream adapter protein MyD88 and nuclear factor κB (NF-κB) that leads to production of pro-inflammatory cytokines. It is especially important in NCC pathogenesis as TLR4 Asp299Gly and Thr399Ile allele was found to be significantly correlated with the incidence and progression to symptomatic NCC [32] . This TLR4 activation is important for macrophages to mount its effector function

as its activation leads to inflammatory cytokines and ROS production [33] while diminished TLR4 leads to M2 polarization of macrophages. The THP-1 derived macrophages treated with ESPs had shown increased expression of all TLRs (1-9) at transcription level but surprisingly we saw no difference in TLR4 expression at translation level. This suggested the post-transcription regulation of TLR4 and this less TLR4 is also making macrophage polarized to M2 and supported our observation of higher expression of M2 macrophage markers. The TLR4 also plays a crucial role in Akt activation by conversion of Akt to active pAkt by activating PI3K [51]. So, we looked for Akt activity and as expected we found down regulation of Akt activity in the presence of chemo-attractant or *E. coli* in ESPs treated macrophages compared to control or LPS stimulated cells (**Figure 5**). Earlier studies had shown Akt activity to be directly associated with ROS production [27,52] hence, we also investigated whether the reduced Akt can lead to alteration in superoxide production in ESPs treated macrophages and we found significantly less ROS in ESPs treated cells (**Figure 6**). A variety of Reactive Oxygen Species (ROS), like H_2O_2 etc., are synthesized and released to the extracellular space and phagocytic vacuoles, where they are known to facilitate destruction of the pathogens [53]. This motivated us to look for the bacterial killing capability of ESPs treated macrophage, and consistent with reduced ROS we found a defect in their bacterial killing capability and when we used chemical Akt activator it restored the phenomenon, thus confirming that reduced killing was due to reduced Akt mediated ROS production.

Of late, there is an increased attention to understanding the mechanisms of the helminth parasite's strong ability of immune modulation. But not much advances have been made in elucidating role of host immune cells miRs during helminthic infections,

especially their role in the development, maturation & specialized function of immune cells [39]. The only literature available on helminths and miRs are about Schistosoma spp, *Fasicola spp* and *Clonorchis sinensis*. The effect of excretory-secretory antigens of *T. crassiceps* on inducing miRs was studied earlier and was found that these antigens repressed LPS-let-7i induction and diminishes inflammatory response in human dendritic cells [54]. Later, role of miR in TcES LPS-induced BMDM was explored and up-regulation of miR-125a-5p, miR-762 & miR-484 was reported. Implying TcES can change post-transcriptional regulation, thereby modulating proinflammatory responses in macrophages [6]. We also did the microarray for miRs in macrophages after ESPs treatment and noticed difference in the expression of miRs and qPCR for these miRs further confirmed upregulation of hsa-miR-19 and down regulation of hsa-miR-125a, hsa-miR-146a & hsa-miR155. Out of these miRs, miR-155, miR-223 & miR-146 have been described for their role in suppressing cytokine signaling & TLRs, and cytokine signaling via a negative feedback regulation loop by down-regulation of IL-1 receptor-associated kinase 1 (IRAK1) and TNF receptor-associated factor 6 (TRAF6) at protein levels [55]. Thus, explaining our other observation of M2 polarization of macrophages and Th2 immune response with ESPs antigen.

This is the first study about the *T. solium* ESPs where they have been studied for their effect on cellular immune responses. We established that ESPs illicit mixed Th1/Th2 cytokines expression and make macrophages polarized towards M2 phenotype. They also affect host miRs expression through which they suppress TLR4 expression, making these treated macrophages suppressed by diminished Akt activation and ROS production thus affecting their bacterial fighting capability. These findings about the role of ESPs on macrophages improves our understanding of host-parasite

interaction and how the Taenia-released molecules cause inflammation. This finding may open avenues for further research aimed at better diagnostic and treatment of NCC. In future, these molecules might find application in other inflammatory and autoimmune disorders. However, to reach that goal it will be imperative to explore the individual antigens identification and their immune modulation mechanisms adapted by them. Our results also suggest that the chronic NCC patients may be prone to other infections and this aspect should be explored by undertaking appropriate clinical study.

Acknowledgements

NA is supported by PhD studentship from the Indian Institute of Technology Mandi. SR acknowledges Dept. of Biotechnology, Gov. of India for financial support. AP acknowledges financial support from Ramalingaswami Fellowship, Department of Biotechnology, Government of India, New Delhi. AP is supported through research grants BT/PR26841/MED/122/121/2017 Department of Biotechnology, Government of India, New Delhi and ECR/2016/000817/LS from SERB, New Delhi.

We sincerely thanks Dr. Meera G Nair, University of California Riverside, USA for her critical review of the manuscript.

CONFLICT OF INTERESTS

None. None of the funders have any role in deciding or influencing the outcome of this study. Here by we declare that we have no conflict of interest and all authors have read and approved the final version of submitted manuscript.

Disclaimer: This manuscript is a part of PhD thesis of Dr Naina Arora, submitted to IIT Mandi.

Availability of data and materials: All relevant data used in this study are included in the manuscript and supplementary files.

References:

[1] Maizels RM, Balic A, Gomez-Escobar N, Nair M, Taylor MD, Allen JE. Helminth parasites - masters of regulation. Immunol Rev. 2004;201(1):89–116. Available from: http://dx.doi.org/10.1111/j.0105-2896.2004.00191.x

[2] Biswas SK, Mantovani A. Macrophage plasticity and interaction with lymphocyte subsets: cancer as a paradigm. Nat Immunol. 2010;11(10):889–96. Available from: http://dx.doi.org/10.1038/ni.1937

[3] Murray PJ, Wynn TA. Protective and pathogenic functions of macrophage subsets. Nat Rev Immunol. 2011;11(11):723–37. Available from: http://dx.doi.org/10.1038/nri3073

[4] Kim SY, Nair MG. Macrophages in wound healing: activation and plasticity. Immunol Cell Biol. 2019;97(3):258–67. Available from: http://dx.doi.org/10.1111/imcb.12236

[5] Maizels RM. Parasitic helminth infections and the control of human allergic and autoimmune disorders. Clin Microbiol Infect. 2016;22(6):481–6. Available from: http://dx.doi.org/10.1016/j.cmi.2016.04.024

[6] Martínez-Saucedo D, Ruíz-Rosado J de D, Terrazas C, Callejas BE, Satoskar AR, Partida-Sánchez S, et al. *Taenia crassiceps*-excreted/secreted products induce a defined MicroRNA profile that modulates inflammatory properties of macrophages. J Immunol Res . 2019;2019:1–24. Available from: http://dx.doi.org/10.1155/2019/2946713

[7] Prasad KN, Prasad A, Gupta RK, Pandey CM, Singh U. Prevalence and associated risk factors of Taenia solium taeniasis in a rural pig farming community of north India. Trans R Soc Trop Med Hyg. 2007;101(12):1241–7. Available from: http://dx.doi.org/10.1016/j.trstmh.2007.04.019

[8] Prasad KN, Prasad A, Verma A, Singh AK. Human cysticercosis and Indian scenario: a review. J Biosci. 2008;33(4):571–82. Available from: http://dx.doi.org/10.1007/s12038-008-0075-y

[9] Prasad KN, Prasad A, Gupta RK, Nath K, Pradhan S, Tripathi M, et al. Neurocysticercosis in patients with active epilepsy from the pig farming community of Lucknow district, north India. Trans R Soc Trop Med Hyg. 2009;103(2):144–50. Available from: http://dx.doi.org/10.1016/j.trstmh.2008.07.015

[10] Del Brutto OH. Neurocysticercosis. In: Handbook of Clinical Neurology. Elsevier; 2014. p. 1445–59. Available from: http://dx.doi.org/10.1016/B978-0-7020-4088-7.00097-3

[11] Taeniasis/cysticercosis. Who.int. [cited 2023 May 3]. Available from: https://www.who.int/news-room/fact-sheets/detail/taeniasis-cysticercosis

[12] Tellez-Zenteno JF, Hernandez-Ronquillo L. Epidemiology of neurocysticercosis and epilepsy, is everything described? Epilepsy Behav. 2017;76:146–50. Available from: http://dx.doi.org/10.1016/j.yebeh.2017.01.030

[13] Garcia HH, White AC, Gilman RH, del Brutto OH, Nash TE, Tsang VCW. New concepts in the diagnosis and management of neurocysticercosis (Taenia solium). Am J Trop Med Hyg . 2005;72(1):3–9. Available from:http://dx.doi.org/10.4269/ajtmh.2005.72.3

[14] Fenwick A. The global burden of neglected tropical diseases. Public Health. 2012;126(3):233–6. Available from: http://dx.doi.org/10.1016/j.puhe.2011.11.015

[15] Arora N, Tripathi S, Kumar P, Mondal P, Mishra A, Prasad A. Recent advancements and new perspectives in animal models for Neurocysticercosis immunopathogenesis. Parasite Immunol. 2017;39(7):e12439. Available from: http://dx.doi.org/10.1111/pim.12439

[16] Arora N, Tripathi S, Sao R, Mondal P, Mishra A, Prasad A. Molecular neuro-pathomechanism of neurocysticercosis: How host genetic factors influence disease susceptibility. Mol Neurobiol. 2018;55(2):1019–25. Available from: http://dx.doi.org/10.1007/s12035-016-0373-6

[17] Del Brutto OH. Human neurocysticercosis: An overview. Pathogens. 2022;11(10):1212. Available from: http://dx.doi.org/10.3390/pathogens11101212

[18] Ma'ayeh SY, Liu J, Peirasmaki D, Hörnaeus K, Bergström Lind S, Grabherr M, et al. Characterization of the Giardia intestinalis secretome during interaction with human intestinal epithelial cells: The impact on host cells. PLoS Negl Trop Dis. 2017;11(12):e0006120. Available from: http://dx.doi.org/10.1371/journal.pntd.0006120

[19] Doonan J, Tarafdar A, Pineda MA, Lumb FE, Crowe J, Khan AM, et al. The parasitic worm product ES-62 normalises the gut microbiota bone marrow axis in inflammatory arthritis. Nat Commun . 2019;10(1). Available from: http://dx.doi.org/10.1038/s41467-019-09361-0

[20] Kaur R, Arora N, Nair MG, Prasad A. The interplay of helminthic neuropeptides and proteases in parasite survival and host immunomodulation. Biochem Soc Trans. 2022;50(1):107–18. Available from: http://dx.doi.org/10.1042/bst20210405

[21] Tsai IJ, The Taenia solium Genome Consortium, Zarowiecki M, Holroyd N, Garciarrubio A, Sanchez-Flores A, et al. The genomes of four tapeworm species reveal adaptations to parasitism. Nature. 2013;496(7443):57–63. Available from: http://dx.doi.org/10.1038/nature12031

[22] Gomez S, Adalid-Peralta L, Palafox-Fonseca H, Cantu-Robles VA, Soberón X, Sciutto E, et al. Genome analysis of Excretory/Secretory proteins in Taenia solium reveals their Abundance of Antigenic Regions (AAR). Sci Rep. 2015;5(1). Available from: http://dx.doi.org/10.1038/srep09683

[23] Arora N, Prasad A. Taenia solium proteins: a beautiful kaleidoscope of pro and anti-inflammatory antigens. Expert Rev Proteomics. 2020;17(7–8):609–22. Available from: http://dx.doi.org/10.1080/14789450.2020.1829486

[24] Arora N, Raj A, Anjum F, Kaur R, Rawat SS, Kumar R, et al. Unveiling Taenia solium kinome profile and its potential for new therapeutic targets. Expert Rev Proteomics. 2020;17(1):85–94. Available from: http://dx.doi.org/10.1080/14789450.2020.1719835

[25] Prasad A, Prasad KN, Kumar GR, Tripathi S, Jha S, Kumar PV, et al. Immune response to different fractions of Taenia solium cyst fluid antigens in patients with neurocysticercosis. Exp Parasitol. 2011;127(3):687–92. Available from: http://dx.doi.org/10.1016/j.exppara.2010.11.006

[26] Singh AK, Singh SK, Singh A, Gupta KK, Khatoon J, Prasad A, et al. Immune response to Taenia solium cysticerci after anti-parasitic therapy. Int J Parasitol. 2015;45(12):749–59. Available from: http://dx.doi.org/10.1016/j.ijpara.2015.05.007

[27] Prasad A, Jia Y, Chakraborty A, Li Y, Jain SK, Zhong J, et al. Inositol hexakisphosphate kinase 1 regulates neutrophil function in innate immunity by

inhibiting phosphatidylinositol-(3,4,5)-trisphosphate signaling. Nat Immunol. 2011;12(8):752–60. Available from: http://dx.doi.org/10.1038/ni.2052

[28] Zhang D, Lee H, Zhu Z, Minhas JK, Jin Y. Enrichment of selective miRNAs in exosomes and delivery of exosomal miRNAs in vitro and in vivo. Am J Physiol Lung Cell Mol Physiol. 2017;312(1):L110–21. Available from: http://dx.doi.org/10.1152/ajplung.00423.2016

[29] Prasad A, Prasad KN, Gupta RK, Pradhan S. Increased expression of ICAM-1 among symptomatic neurocysticercosis. J Neuroimmunol. 2009;206(1–2):118–20. Available from: http://dx.doi.org/10.1016/j.jneuroim.2008.09.015

[30] Mylonas KJ, Nair MG, Prieto-Lafuente L, Paape D, Allen JE. Alternatively activated macrophages elicited by helminth infection can be reprogrammed to enable microbial killing. J Immunol. 2009;182(5):3084–94. Available from: http://dx.doi.org/10.4049/jimmunol.0803463

[31] Mishra BB, Mishra PK, Teale JM. Expression and distribution of Toll like receptors in the brain during Neurocysticercosis. J Neuroimmunol. 2006;181:46–56. Available from: http://dx.doi.org/10.1016/j.jneuroim.2006.07.019

[32] Verma A, Prasad KN, Gupta RK, Singh AK, Nyati KK, Rizwan A, et al. Toll-like receptor 4 polymorphism and its association with symptomatic neurocysticercosis. J Infect Dis . 2010;202(8):1219–25. Available from: http://dx.doi.org/10.1086/656395

[33] Yuan X, Zhou Y, Wang W, Li J, Xie G, Zhao Y, et al. Activation of TLR4 signaling promotes gastric cancer progression by inducing mitochondrial ROS production. Cell Death Dis. 2013;4(9):e794–e794. Available from: http://dx.doi.org/10.1038/cddis.2013.334

[34] Jo H, Mondal S, Tan D, Nagata E, Takizawa S, Sharma AK, et al. Small molecule-induced cytosolic activation of protein kinase Akt rescues ischemia-elicited neuronal death. Proc Natl Acad Sci USA. 2012;109(26):10581–6. Available from: http://dx.doi.org/10.1073/pnas.1202810109

[35] Wu D, Yu D, Wang X, Yu B. F- actin rearrangement is regulated by mTORC 2/Akt/Girdin in mouse fertilized eggs. Cell Prolif. 2016;49(6):740–50. Available from: http://dx.doi.org/10.1111/cpr.12285

[36] Bartel DP. MicroRNAs: Target recognition and regulatory functions. Cell. 2009;136(2):215–33. Available from: http://dx.doi.org/10.1016/j.cell.2009.01.002

[37] Molnar A, Melnyk CW, Bassett A, Hardcastle TJ, Dunn R, Baulcombe DC. Small silencing RNAs in plants are mobile and direct epigenetic modification in recipient cells. Science. 2010;328(5980):872–5. Available from: http://dx.doi.org/10.1126/science.1187959

[38] Krol J, Loedige I, Filipowicz W. The widespread regulation of microRNA biogenesis, function and decay. Nat Rev Genet. 2010;11(9):597–610. Available from: http://dx.doi.org/10.1038/nrg2843

[39] Arora N, Tripathi S, Singh AK, Mondal P, Mishra A, Prasad A. Micromanagement of immune system: Role of miRNAs in helminthic infections. Front Microbiol. 2017;8. Available from: http://dx.doi.org/10.3389/fmicb.2017.00586

[40] Licursi V, Conte F, Fiscon G, Paci P. MIENTURNET: an interactive web tool for microRNA-target enrichment and network-based analysis. BMC Bioinformatics. 2019;20(1). Available from: http://dx.doi.org/10.1186/s12859-019-3105-x

[41] Hou C, Chen Y, Huang X, Huang Q, Li M, Tan X. miR-19 targets PTEN and mediates high mobility group protein B1(HMGB1)-induced proliferation and migration of human airway smooth muscle cells. PLoS One. 2019;14(6):e0219081. Available from: http://dx.doi.org/10.1371/journal.pone.0219081

[42] Zhou C, Zhao L, Wang K, Qi Q, Wang M, Yang L, et al. MicroRNA-146a inhibits NF-κB activation and pro-inflammatory cytokine production by regulating IRAK1 expression in THP-1 cells. Exp Ther Med. 2019; Available from: http://dx.doi.org/10.3892/etm.2019.7881

[43] Reyes JL, Terrazas CA, Vera-Arias L, Terrazas LI. Differential response of antigen presenting cells from susceptible and resistant strains of mice to Taenia crassiceps infection. Infect Genet Evol. 2009;9(6):1115–27. Available from: http://dx.doi.org/10.1016/j.meegid.2009.05.011

[44] Brandt JRA, Geerts S, Deken RD, Kumar V, Ceulemans F, Brijs L, et al. A monoclonal antibody-based ELISA for the detection of circulating excretory-secretory antigens in Taenia saginata cysticercosis. Int J Parasitol. 1992;22(4):471–7. Available from: http://dx.doi.org/10.1016/0020-7519(92)90148-e

[45] Spolski, Thomas, See, Mooney, Kuhn. Larval Taenia crassiceps secretes a protein with characteristics of murine interferon-γ. Parasitol Res. 2002;88(5):431–8. Available from: http://dx.doi.org/10.1007/s00436-002-0590-y

[46] Terrazas CA, Alcántara-Hernández M, Bonifaz L, Terrazas LI, Satoskar AR. Helminth- excreted/secreted products are recognized by multiple receptors on DCs to block the TLR response and bias Th2 polarization in a cRAF dependent

pathway. FASEB J. 2013;27(11):4547–60. Available from: http://dx.doi.org/10.1096/fj.13-228932

[47] Chauhan A, Sun Y, Pani B, Quenumzangbe F, Sharma J, Singh BB, et al. Helminth induced suppression of macrophage activation is correlated with inhibition of calcium channel activity. PLoS One. 2014;9(7):e101023. Available from: http://dx.doi.org/10.1371/journal.pone.0101023

[48] Ley K. M1 means kill; M2 means heal. J Immunol. 2017;199(7):2191–3. Available from: http://dx.doi.org/10.4049/jimmunol.1701135

[49] Takeuchi O, Akira S. Pattern recognition receptors and inflammation. Cell. 2010;140(6):805–20. Available from: http://dx.doi.org/10.1016/j.cell.2010.01.022

[50] Mishra BB, Gundra UM, Teale JM. Expression and distribution of Toll-like receptors 11–13 in the brain during murine neurocysticercosis. J Neuroinflammation. 2008;5(1). Available from: http://dx.doi.org/10.1186/1742-2094-5-53

[51] Monick MM, Carter AB, Robeff PK, Flaherty DM, Peterson MW, Hunninghake GW. Lipopolysaccharide activates Akt in human alveolar macrophages resulting in nuclear accumulation and transcriptional activity of β-catenin. J Immunol. 2001;166(7):4713–20. Available from: http://dx.doi.org/10.4049/jimmunol.166.7.4713

[52] Bäumer AT, Freyhaus H, Sauer H, Wartenberg M, Kappert K, Schnabel P, et al. Phosphatidylinositol 3-kinase-dependent membrane recruitment of rac-1 and p47phox is critical for α-platelet-derived growth factor receptor-induced production of reactive oxygen species. J Biol Chem. 2008;283(12):7864–76. Available from: http://dx.doi.org/10.1074/jbc.m704997200

[53] Dharmaraja AT. Role of Reactive Oxygen Species (ROS) in Therapeutics and Drug Resistance in Cancer and Bacteria. J Med Chem. 2017;60(8):3221–40.

[54] Terrazas LI, Sánchez-Muñoz F, Pérez-Miranda M, Mejía-Domínguez AM, Ledesma-Soto Y, Bojalil R, et al. Helminth excreted/secreted antigens repress expression of LPS-induced let-7i but not miR-146a and miR-155 in human Dendritic Cells. Biomed Res Int. 2013;2013:1–6. Available from: http://dx.doi.org/10.1155/2013/972506

[55] Taganov KD, Boldin MP, Chang K-J, Baltimore D. NF-κB-dependent induction of microRNA miR-146, an inhibitor targeted to signaling proteins of innate immune responses. Proc Natl Acad Sci USA. 2006;103(33):12481–6. Available from: http://dx.doi.org/10.1073/pnas.0605298103

Supplementary Information

Supplement Figure 1: Flow chart outlining the methodology

Supplement Figure 2: Cysts infested pork from naturally infected swine (a) and isolated cyst (b)

Supplement Figure 3: Species identification of Taenia by PCR, all our samples were positive only for T. solium and gave 984bp product specific for this species

Supplement Figure 4: SDS-PAGE and silver staining of T. solium ESPs

Supplement Figure 5: Quantification of progression of secreted cytokines by ELISA

Supplement Figure 6: Apoptosis assay. The ESP treated cells showed no sign of apoptosis as compared to positive control

Supplement Figure 7 Caspase activity. The ESP treated cells showed no sign of caspase 3 as compared to positive control

Supplementary Table 1: List of primers used in the study

Supplementary Table 2: List of primers used for miRNA validation in the study

Click here to access/download
Supporting Information
Suplement.docx

Click here to access/download
Supporting Information
GA_affy_CHP-3.xls
