# Supplementary material for: Taenia solium excretory secretory proteins (ESPs) suppresses TLR4/AKT mediated ROS formation in human macrophages via hsa-miR-125
Source: PLoS Negl Trop Dis. 2023 Dec 29;17(12):e0011858. doi: 10.1371/journal.pntd.0011858 (PMC10783723; doi:10.1371/journal.pntd.0011858)
Supplement: Attachment — Submitted filename: Comments_ROS.docx [file pntd.0011858.s006.docx]

[1] A letter containing a detailed list of your responses to the review comments and a description of the changes you have made in the manuscript. Please note while forming your response, if your article is accepted, you may have the opportunity to make the peer review history publicly available. The record will include editor decision letters (with reviews) and your responses to reviewer comments. If eligible, we will contact you to opt in or out.

[2] Two versions of the revised manuscript: one with either highlights or tracked changes denoting where the text has been changed; the other a clean version (uploaded as the manuscript file).

Important additional instructions are given below your reviewer comments.

Please prepare and submit your revised manuscript within 60 days. If you anticipate any delay, please let us know the expected resubmission date by replying to this email. Please note that revised manuscripts received after the 60-day due date may require evaluation and peer review similar to newly submitted manuscripts.

 
Thank you again for your submission. We hope that our editorial process has been constructive so far, and we welcome your feedback at any time. Please don't hesitate to contact us if you have any questions or comments.

 
Sincerely,

Andrew Scott MacDonald
Academic Editor
PLOS Neglected Tropical Diseases

Cinzia Cantacessi
Section Editor
PLOS Neglected Tropical Diseases

***********************

Reviewer's Responses to Questions

<b>Key Review Criteria Required for Acceptance?</b></br></br>
As you describe the new analyses required for acceptance, please consider the following:</br></br>

<b>Methods</b></br></br>
-Are the objectives of the study clearly articulated with a clear testable hypothesis stated?</br>
-Is the study design appropriate to address the stated objectives?</br>
-Is the population clearly described and appropriate for the hypothesis being tested?</br>
-Is the sample size sufficient to ensure adequate power to address the hypothesis being tested?</br>
-Were correct statistical analysis used to support conclusions?</br>
-Are there concerns about ethical or regulatory requirements being met?</br></br>

Reviewer #1: The study design is not appropriate for testing the hypothesis. The population size is not clearly described, nor does it seem sufficient.

Reply: We would like to humbly state that this study is not about population study hence the comment is not valid. Above that, all the experiments were performed thrice with technical triplicates as per the standard norms.

Reviewer #2: -Are the objectives of the study clearly articulated with a clear testable hypothesis stated?
Yes
-Is the study design appropriate to address the stated objectives?

Mostly
Reply: Thanks for understanding the study design and validating our material and methods.
--------------------

<b>Results</b></br></br>
-Does the analysis presented match the analysis plan?</br>
-Are the results clearly and completely presented?</br>
-Are the figures (Tables, Images) of sufficient quality for clarity?</br></br>

Reviewer #1: The figures are not of sufficient clarity for interpretation, and the legend do not offer enough details on the experimental design. The flow of the figures do not match the flow of the text, which can be confusing for the reader.

Reply: We have redrafted most of the figure legends as per the comments by the reviewer. Hopefully you will find them of more informative now.

Reviewer #2: -Does the analysis presented match the analysis plan?

Yes
-Are the results clearly and completely presented?

Mostly

-Are the figures (Tables, Images) of sufficient quality for clarity?

Mostly
Reply: We appreciate the reviewer for taking time and going through our manuscript. Thanks for understanding the study design and validating our material and methods.
--------------------

<b>Conclusions</b></br></br>
-Are the conclusions supported by the data presented?</br>
-Are the limitations of analysis clearly described?</br>
-Do the authors discuss how these data can be helpful to advance our understanding of the topic under study?</br>
-Is public health relevance addressed?</br></br>

Reviewer #1: The conclusions are not supported by the data presented.

Reply: We have included all the suggestions from the reviewers in the modified manuscript, and hopeful that you will like this modified version.

Reviewer #2: -Are the conclusions supported by the data presented?
Yes

-Do the authors discuss how these data can be helpful to advance our understanding of the topic under study?

Yes
Reply: We appreciate the reviewer for taking time and going through our manuscript and agreeing that our study give meaningful addition to the existing information to the filed.

--------------------

<b>Editorial and Data Presentation Modifications?</b></br><br/>
Use  this section for editorial suggestions as well as relatively minor modifications of existing data that would enhance clarity. If the only modifications needed are minor and/or editorial, you may wish to recommend “Minor Revision” or “Accept”.

Reviewer #1: (No Response)

Reviewer #2: (No Response)

--------------------

<b>Summary and General Comments</b></br></br>
Use  this section to provide overall comments, discuss strengths/weaknesses of the study, novelty, significance, general execution and scholarship. You may also include additional comments for the author, including concerns about dual publication, research ethics, or publication ethics. If requesting major revision, please articulate the new experiments that are needed.

Reviewer #1: This manuscript address the role of ESPs in macrophage modulation. It is currently unclear if ESPs are indeed affecting polarization with the present datasets. Of note, it might be due to a poor presentation of the experimental design. The authors argue for a TLR4 reduction of ROS production through a miR dependent mechanism. However, if my understanding of the experimental design is correct ESP on its own is not a TLR4 agonist (as many other helminth products) and as such do not cause Akt phosphorylation. I failed to see how this is a decrease of anything ? If ESP combined with LPS, caused a decrease in Akt phosphorylation then the mechanism would make sense, as ESP seems to decreasing phagocytosis.
Please find detail comment sin the attached pdf.

Reply: This is a very surprising comment but, plz see few references where the helminths products/soluble etc was found to be directly interfering with TLR4

1. Ottow MK, Klaver EJ, van der Pouw Kraan TC, Heijnen PD, Laan LC, Kringel H, Vogel DY, Dijkstra CD, Kooij G, van Die I. The helminth Trichuris suis suppresses TLR4-induced inflammatory responses in human macrophages. Genes Immun. 2014 Oct;15(7):477-86. doi: 10.1038/gene.2014.38. Epub 2014 Jul 10. PMID: 25008860.
2. Semnani RT, Venugopal PG, Leifer CA, Mostböck S, Sabzevari H, Nutman TB. Inhibition of TLR3 and TLR4 function and expression in human dendritic cells by helminth parasites. Blood. 2008 Aug 15;112(4):1290-8. doi: 10.1182/blood-2008-04-149856. Epub 2008 Jun 9. PMID: 18541719; PMCID: PMC2515123.
3. Pan Q, Zhang Q, Chu J, Pais R, Liu S, He C, Eko FO. *Chlamydia abortus* Pmp18.1 Induces IL-1β Secretion by TLR4 Activation through the MyD88, NF-κB, and Caspase-1 Signaling Pathways. Front Cell Infect Microbiol. 2017 Dec 18;7:514. doi: 10.3389/fcimb.2017.00514. PMID: 29326885; PMCID: PMC5741698.

Reviewer #2: The manuscript of Arora N et al. describes the function of Taenia solium excretory secretory proteins (ESPs) to supress TLR4/AKT ROS formation in human macrophages via has-miR-125. This study adds invaluable value to the field of how helminth can suppress host immune response to establish the infections.

Reply: Thanks for reading our manuscript and finding its potential.

Major comments:

1. There are two bands of TLR4 in Figure 7D while Figure 4 has only one band, can authors please explain and show the full blot with labelling?

Reply: Thanks for your observation and comment. We have improved the all-western blot data in the light of your comments. And for the ease of understanding to the readers.

However, the differences in the observed TLR4 bands between Figure 7D and Figure 4 are indeed due to variations in glycosylation levels of the TLR4 protein. TLR4, being a glycoprotein, can undergo post-translational modifications, including glycosylation, which can result in multiple bands of varying molecular weights on a Western blot.The phenomenon of multiple TLR4 bands in Western blot analysis is well-documented and has been attributed to differences in glycosylation patterns. Glycosylation involves the addition of carbohydrate molecules to proteins, leading to variations in protein mobility during electrophoresis. These modifications can affect the apparent size and mobility of the protein bands on the gel.

For a more detailed and conclusive explanation of the specific glycosylation patterns observed in our study, we have consulted the primary antibody used in our experiments. The primary antibody employed, Toll-Like Receptor 4 (D8L5W) Rabbit Monoclonal Antibody, recognizes TLR4 and its isoforms, taking into account the variations in glycosylation.. (<https://www.cellsignal.com/products/primary-antibodies/toll-like-receptor-4-d8l5w-rabbit-mab/14358>) (doi: 10.1074/jbc.M109910200.)

1) Akt inhibitor should be included as a control group.

Reply: The introduction of an Akt inhibitor could result in non-specific outcomes that are unrelated to TLR4 suppression, making it challenging to attribute any observed effects specifically to ESP. It is important to maintain the specificity of experimental controls to isolate the impact of the variable being studied. In this case, we aim to investigate the role of ESP in TLR4 suppression. To achieve this, we would ideally use a control group that specifically targets the TLR4 signaling pathway, rather than a broad inhibitor like Akt, which affects multiple pathways. Using an Akt inhibitor may obscure the direct effects of ESP on TLR4.

2) If the Taenia solium does affect microglia, a microglia cell line such as HMC3 could be used

Reply: This is indeed a very good suggestion. and Microglia are indeed specialized cells with a crucial role in the central nervous system. However, the scope of our current study was intentionally limited to human macrophages only. Our long-term objective is to identify potential biomarkers in circulation that could be indicative of host responses to Taenia solium infection.

One of the key considerations in our study design is the fact that excretory-secretory products (ESP) from Taenia solium are secreted by the parasite and can potentially reach the bloodstream. Additionally, during the pathogenesis of neurocysticercosis (NCC), the blood-brain barrier can become compromised, leading to increased permeability. As such, our primary interest was to investigate the systemic effects of ESP on host immune cells, particularly human macrophages, which play a pivotal role in the innate immune response.

3. In 3.5.,
a miR-125 inhibitor could be added as miR-125 is suspected to be critical for the TLR4 and AKT expression

Reply: We appreciate your thoughtful recommendation and have taken it into account as we continue to unravel the intricate mechanisms underlying our research.

Our initial miRNA microarray analysis indeed indicated a downregulation of miR-125. This finding was subsequently validated through quantitative PCR (qPCR), strengthening the evidence for miR-125's differential expression in our experimental context. Given its downregulated status, we proceeded to investigate the functional implications of miR-125 through the introduction of a miR-125 mimic.

The rationale behind employing a miR-125 mimic was to explore whether the exogenous supplementation of miR-125 could potentially rescue the suppressed activities we observed in our study. This approach allows us to probe the specific effects of miR-125 in the context of TLR4 and AKT expression and evaluate its potential as a regulatory factor in these pathways.

Minor comments:

1. methods do not provide details. please check all methods section
e.g.
Line 139:  name of antibiotics and concentration are missing?

Added : 1% antibiotic-antimycotic (#[15240096](https://www.thermofisher.com/order/catalog/product/15240096),Gibco, Invitrogen, USA)

Line 147 how to differentiate the THP-1 into macrophages is missing

Added: using10 ng/mL phorbol 12-myristate 13-acetate (PMA) (Sigma-Aldrich, USA) for 24 hrs, followed by a 24-hr rest period

Line 219 how RIN is measured (by Bioanalyzer?), which kit?

2100 Bioanalyzer Instrument (#G2939BA, Agilent) (small RNA chip #5065-4413)


2. some improper terms/phase, please check throughout the manuscripts
e.g. line 252 cell culture soup and line 279  give … cytokines

3. methods and results are mixed in the results section

Comments in PDF:

1. Line 82 “ already defined previously”

corrected

2. Line 101 wrong structure of the sentence

edited

3. Line 290 It would be interesting to get the concentration for the cytokines measured by flow cytometry rather than relative expression.

To what is it normalized ?

Figure 2 represents cytokine quantification done on flow cytometer using cell bead assay. The normalization in figure 1 for relative expression is done using actin

4. Line 329 This should not really come as a surprise and might actually be true for many TLRs, what would be more relevant to report is signaling activation of those TLRs, especially because activation of TLRs by ES products is poorly characterized, and only a few examples have been reported to date.

Edited

5. Line 380 The effect is quite small and poorly convincing here. It might be useful to do a titration of ESP to establish the there is indeed a decrease in phosphorylation of Akt.
6. Line 383 A control AKT Activator without ESP is missing here

PMID: 32762278 "It is believed that AKT pathway is conserved to enhance the phagocytic activity of macrophages and pathogens adopt strategies/mechanism to suppress AKT activity and evade host response." Since phagocytic activity is dependent on AKT activation, a positive control was not added, the aim of experiment was to demonstrate rescue of AKT on addition on AKT activator.

7. Line 396 less ROS than what ?

edited

8. Line 397 Is the left part of A a representative results from the right part ? On how many samples has this been generated ? There is no error bars on the graph and the data as it stands are poorly convincing.

All the experiments are representation of results from three independent experiments. The figure legend has been updated for clarity, left is histogram representation of data on right. The image has been updated to add the error bars.

9. Line 399 On how many samples is this done ? Are the macrophages treated only with ESP or with ESP and a ROS activator ? To me it just look like the data show that the ESP are not a ROS activator
10. All the experiments are representation of results from three independent experiments. And the. “To me it just look like the data show that the ESP are not a ROS activator” that’s what we wanted to claim that ESP are suppressing/not generating ROS when macrophages are treated with them.

11. Line 421 On this blot, the inhibition of pAkt seems to be much stronger than on previous figure, which is true. Please pick more representative images.

Thanks for the observation. We had changed the blot.

12. Line 421 Have you assessed the survival of macrophages after treatment with increasing dose of ESP ? You could be looking at toxicity only here

To rule out toxicity related to ESP, apoptosis assay and caspase 3 activity were assessed for ESP treated macrophages, the data is provided in supplementary figure 6 & 7

13. Line 426 Which function is measured here?

Edited
