# Supplementary material for: Taenia solium excretory secretory proteins (ESPs) suppresses TLR4/AKT mediated ROS formation in human macrophages via hsa-miR-125
Source: PLoS Negl Trop Dis. 2023 Dec 29;17(12):e0011858. doi: 10.1371/journal.pntd.0011858 (PMC10783723; doi:10.1371/journal.pntd.0011858)
Supplement: Attachment — Submitted filename: Clarification to reviwers.docx [file pntd.0011858.s007.docx]

Reviewer #1: As previously stated, it is difficult to follow the experimental design of the authors. Some parts of the methods are not described either in the method section or in the figure legend (For example use of fMLP, or SC79 mentioned in the main result section but neither in figure nor in the material and methods).

**Clarification**: Dear Reviewer, once again thanks a lot for giving your valuable time to go through our modified manuscript and accepting most of the changes which had been carried out in the light of your constructive suggestions. Thank you.

As per your suggestion we had carried out major changes in the flow of the manuscript and asked few of our senior collaborators and research scholar to read and give their opinion. Mostly they were satisfied and hopefully you will also be satisfied with the flow of manuscript this time.

**Comments:** The text do not follow the order of the figures and those are not always cited in the text (For example which figure represent the SC79 treatment?) . It is incredibly complicated to review the article in those conditions and the authors have completely ignored my previous comment. 
Instead of clarifying the experimental design and the associated text of figure 5 and 6 as requested, the authors answered with a list of publications and a less than pleasant comment.

**Clarification**: At first our apologies if by any chance you felt hurt due to any of our comments which was never intended to hurt any one and specially person who is giving time to go through our manuscript and appreciating the research.

In the modified manuscript we had mentioned fMLP, SC79 treatment with suitable reference too. We hope this time you will not have any issue to go through the manuscript

**Comments:** Interestingly the paper cited, do indeed address my question and have the clear experimental design I was suggesting to the authors of the current manuscript to embrace. For example, Figure 1 of <https://www.nature.com/articles/gene201438> actually compare LPS only versus LPS+worm extract versus worm extract only. Which is exactly what I was asking the authors with my previous comment.

**Clarification**: Thanks for suggesting this paper and we followed as per your suggestion. We are happy that you are satisfied with this modification.

**Comments:** Overall, what I can judge from the paper is of excellent quality and certainly worse reporting to the community, however ignoring comments from reviewer is extremely rude and certainly not acknowledging the time freely given to this review process.
**Clarification**: Dear reviewer thanks for appreciating the science behind the manuscript. We had carried out extensive modifications in the light of your comments and hope that this will be acceptable to you too. One again, thanks for understanding in admiring our science.

Reviewer #2: (No Response)
